# Abrupt changes in algal biomass of thousands of US lakes are related to climate and are more likely in low-disturbance watersheds

**DOI:** 10.1073/pnas.2416172122

**Published:** 2025-02-24

**Authors:** Patricia A. Soranno, Patrick J. Hanly, Katherine E. Webster, Tyler Wagner, Andrew McDonald, Arnab Shuvo, Erin M. Schliep, Kaitlin L. Reinl, Ian M. McCullough, Pang-Ning Tan, Noah R. Lottig, Kendra Spence Cheruvelil

**Affiliations:** ^a^Department of Integrative Biology, Michigan State University, East Lansing, MI 48864; ^b^Department of Fisheries and Wildlife, Michigan State University, East Lansing, MI 48864; ^c^Ecology, Evolution, and Behavior Program, Michigan State University, East Lansing, MI 48864; ^d^United States Geological Survey, Pennsylvania Cooperative Fish and Wildlife Research Unit, The Pennsylvania State University, University Park, PA 16802; ^e^Department of Computer Science and Engineering, Michigan State University, East Lansing, MI 48864; ^f^Hasler Laboratory of Limnology, University of Wisconsin-Madison, Madison, WI 53706; ^g^Department of Statistics, North Carolina State University, Raleigh, NC 27607; ^h^Lake Superior National Estuarine Research Reserve, University of Wisconsin-Madison Division of Extension, Superior, WI 54880; ^i^Trout Lake Station, University of Wisconsin-Madison, Boulder Junction, WI 54512; ^j^Lyman Briggs College, Michigan State University, East Lansing, MI 48864

**Keywords:** lake algal biomass, climate change, time series modeling, abrupt ecological change, temporal ecology

## Abstract

Climate change is predicted to intensify lake algal blooms globally and result in regime shifts. However, observed increases in algal biomass do not consistently correlate with air temperature or precipitation, and evidence is lacking for a causal effect of climate or the nonlinear dynamics needed to demonstrate regime shifts. We modeled the causal effects of climate on annual lake chlorophyll (a measure of algal biomass) over 34 y for 24,452 lakes across broad ecoclimatic zones of the United States and evaluated the potential for regime shifts. We found that algal biomass was causally related to climate in 34% of lakes. In these cases, 71% exhibited abrupt but mostly temporary shifts as opposed to persistent changes, 13% had the potential for regime shifts. Climate was causally related to algal biomass in lakes experiencing all levels of human disturbance, but with different likelihood. Climate causality was most likely to be observed in lakes with minimal human disturbance and cooler summer temperatures that have increased over the 34 y studied. Climate causality was variable in lakes with low to moderate human disturbance, and least likely in lakes with high human disturbance, which may mask climate causality. Our results explain some of the previously observed heterogeneous climate responses of lake algal biomass globally and they can be used to predict future climate effects on lakes.

## Significance

Algal biomass has been increasing in many lakes in recent years. These increases are thought to be due to climate change, potentially leading to regime shifts (large ecosystem responses to small disturbances that push past a tipping point). However, evidence for these expectations remains scarce. To address this gap, we analyzed 24,452 lake time series across 34 y. We found that climate caused changes in algal biomass in a third of the lakes, but only 13% had the potential for regime shifts, and just 4% showed increased algae through time. Instead, most lakes (71%) showed abrupt but temporary algal biomass changes, with a higher likelihood of a climate response under certain environmental conditions with low to moderate human disturbance.

Primary production of the biosphere is influenced by climate. However, the causal effects of climate on primary production in oceans, inland waters, grasslands, arid lands, and forests have been challenging to quantify at broad scales (1–4). Quantifying such relationships is essential to better inform the inclusion of primary producers in earth system models (5) and to identify the likelihood of regime shifts, which can cause shifts to new stable states that are resistant to management actions. Increasingly, scientists use proxies for primary productivity from satellite imagery to quantify standing stocks or biomass at broader scales of space and time (2, 4–6). However, even with this increased access to frequent biomass time series, quantifying the causal effects of climate on biomass at macroscales is challenging. Here, we study the effects of climate on the algal biomass of 24,452 inland lakes across three decades and demonstrate an analytical approach that can be applied to other biosphere components at continental to global scales using historical records from remote sensing platforms.

Several challenges limit the ability to document the causal relationships between climate and lake algal biomass or the nonlinear dynamics needed to demonstrate climate-caused regime shifts (6–10). First, although average global air temperature and extreme weather events have changed substantially in the last half-century, natural variability in temperature and precipitation at regional and local scales makes it challenging to quantify general ecosystem responses to climate, especially at decadal timescales (3, 8). Second, the temporal dynamics of lake algae are controlled by a complex mix of lake, watershed, and environmental factors that vary across individual lakes and that can mediate algal response to climate (e.g., lake depth, water temperature, land use/cover, soils, inflows of water and nutrients, food web dynamics) (11–16). Third, the human land use footprint, particularly agriculture, has increased in spatial extent and intensified in many locations (12, 17–19), which may overwhelm and mask climate effects on lake algae. Fourth, documentation of abrupt changes alone is not sufficient to quantify climate-caused regime shifts, since there are other contributing factors which require sufficient data and appropriate approaches to document (10, 20). Finally, at least for grasslands, primary productivity responses to climate are nonlinear, so linear approaches cannot detect the relationships (4). These challenges have limited our ability to define and quantify the influence of climate on large numbers of diverse ecosystems.

Scientists can now fill this gap for inland waters by conducting broad-scale studies that incorporate diverse environmental context data and harness long-term data for in-lake algal biomass data from satellite sensors that enable robust time series analyses. By combining such models with recent theoretical advancements in temporal ecology, it is possible to make inferences from driver–response relationships at broad scales (9, 21–24). In this study, we ask two questions: 1) What are the causal effects of climate on lake algal biomass, and is there a potential for regime shifts? 2) How is climate causality of algal biomass related to lake temporal patterns and environmental context?

We analyzed 34-y time series (1985-2018) of validated, satellite-derived algal biomass for 24,452 lakes that are representative of the full range of environmental characteristics of the conterminous US, including small lakes (≥4 ha) that have only rarely been examined at these scales (25, 26). We measured annual algal biomass as the median lake chlorophyll-a (CHL) concentration from a minimum of eight observations collected between April and October for each year. We quantified relationships between the CHL time series and 16 climate metrics [temperature, precipitation, drought, and El Niño Southern Oscillation (ENSO) for four time periods].

**Fig. 1.**
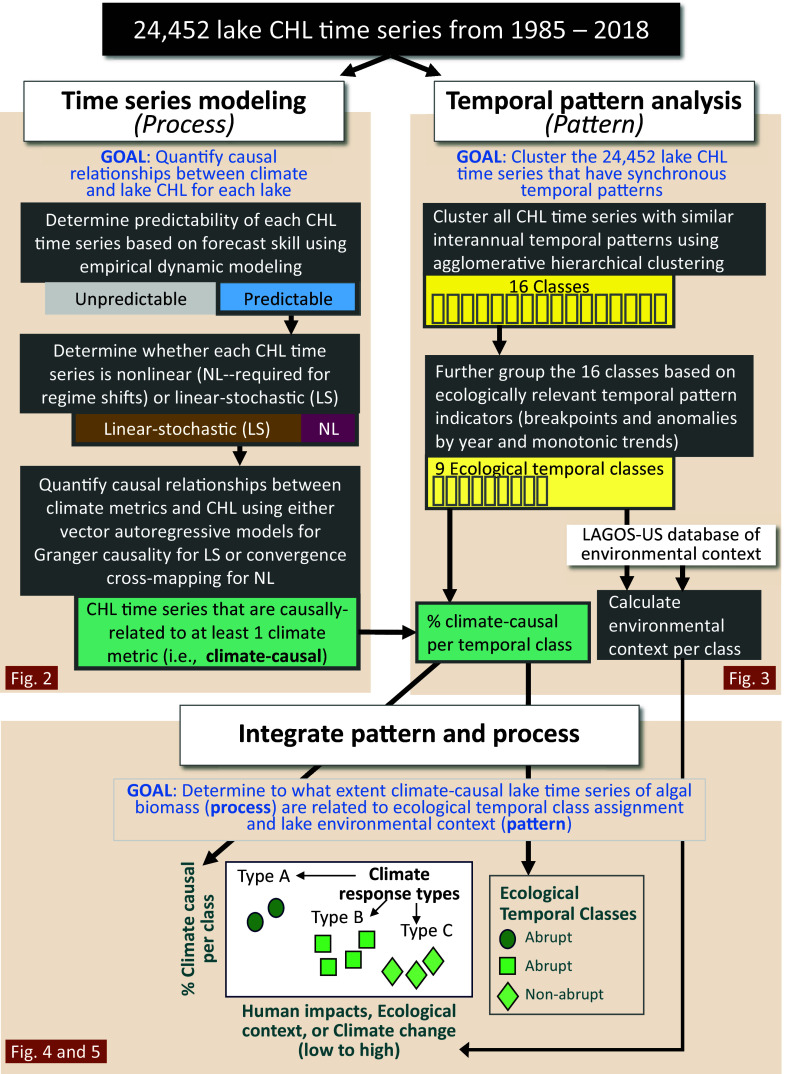
Overview of the approach to analyze thousands of time series to estimate climate effects on algal biomass (CHL). The first box (*Top Left*) illustrates the time series modeling, which analyzes the temporal “process” to examine the causal effect of climate on lake CHL. The second box (*Top Right*) illustrates the temporal pattern analysis which calculates temporal “patterns” of lake CHL, which are indicators of temporal changes over time. The third box (*Bottom*) illustrates the integration of temporal pattern and process to examine the environmental contexts where climate is more likely to be causally related to algal biomass.

**Fig. 2.**
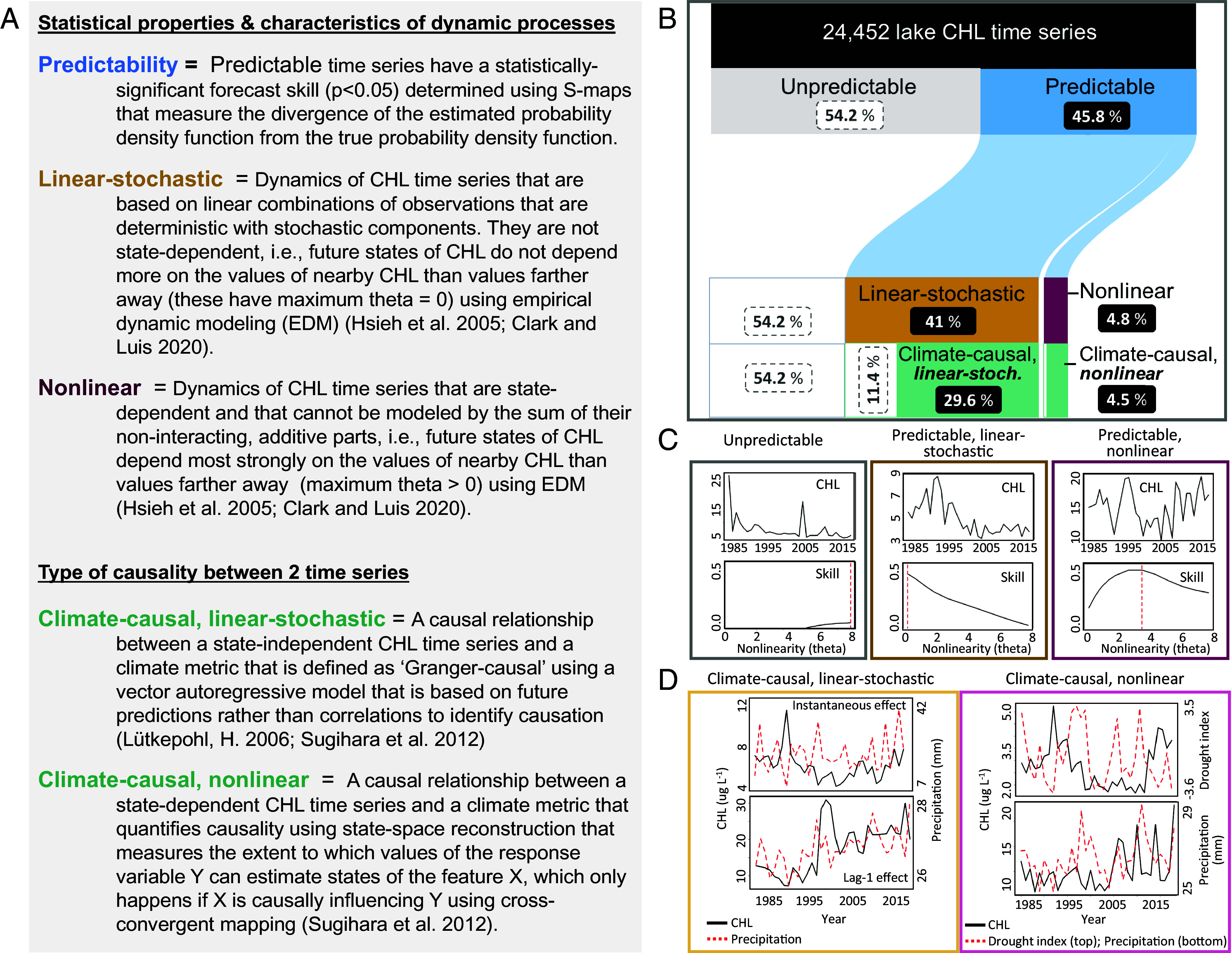
Definitions and example results for the time series modeling that includes the lake CHL time series that are causally related to climate. (*A*) Definitions of key terms. (*B*) A Sankey plot that shows results from the time series modeling, including the percentage of CHL time series with statistical properties related to predictability (gray or blue), linear-stochastic vs. nonlinearity (dark gold or purple), and climate-causality (green). (*C*) Example plots from three lakes showing the CHL time series (CHL) and the predictive skill vs. theta (Skill) from the empirical dynamic modeling analysis for the three statistical properties. (*D*) Example plots from four lakes for CHL and a climate metric time series causally related to the CHL time series from 1985 to 2018. For the linear-stochastic time series, the example lakes have a significant relationship between climate and lake CHL with either instantaneous causality (*Top*) or lag-1 Granger causality (*Bottom*). For the nonlinear time series, both example lakes have significant causal relationships between climate and lake CHL.

Our integrated analytical approach includes three major components (Fig. 1). First, to quantify causal relationships between climate and CHL, we used two types of time series modeling approaches, based on the statistical properties of the time series for each lake (see Fig. 2*A* for definitions). We first calculated the predictability of CHL time series, defined as a time series with significant forecast skill distinguishing it from a purely stochastic process (27). For the predictable CHL time series, we used empirical dynamic modeling to determine whether the lake time series was nonlinear (required to potentially lead to regime shifts) or linear-stochastic (10, 27). We then modeled climate causality of CHL time series using convergence cross-mapping (4, 28) for nonlinear time series and vector autoregressive models for Granger causality (29) for linear-stochastic time series. The second component of our approach was temporal pattern analysis, using a machine learning algorithm to cluster all CHL time series into 16 classes that we further refined to nine ecological temporal classes with synchronous temporal patterns and ecologically relevant indicators of change (30): abrupt and persistent, abrupt but temporary, monotonic, and no pattern (24). Third, to answer our second research question, we synthesized these two analytical streams to integrate temporal pattern and process by examining the relationship between climate-causal lake time series and ecological temporal class assignment and lake environmental context.

The reciprocal relationship between pattern and process is a long-standing and flexible concept in landscape ecology that has improved the understanding of spatial ecology (31). However, the interplay of pattern and process for temporal ecology has been understudied (9, 24) and is yet to be integrated with spatial patterns of environmental context at broad spatial extents. The growing availability of spatial and temporal data, through the maturation of historical remote sensing platforms, offers an exciting opportunity to integrate spatial and temporal ecology with pattern and process.

## Results and Discussion

### Climate Causality and Regime Shifts.

Climate time series were causally related to lake CHL time series (i.e., climate-causal) in 34% of the 24,452 US lakes tested (Fig. 2*B*). The remainder of the CHL time series were either unpredictable (54%) or predictable but not related to any of the climate metrics tested (12%). Our results support the expectation that climate change can influence lake CHL (32–34), but not in all lakes and with differing effects (Fig. 2 *C* and *D*). In addition, only 4.5% of all climate-causal time series were nonlinear. This result implies that the potential for climate change to lead to regime shifts [sensu (10, 27)] in lake CHL is limited, consistent with a recent study of 1,015 global lakes (6). Our broad-scale results establish a benchmark for predicting how lakes have responded to climate in the recent past while recognizing that these patterns and relationships could change under different climate change trajectories.

For the 8,352 climate-causal lakes, the relationships between climate and lake CHL were heterogeneous across regions and climate metrics, and the directionality and the climate-seasonality of the effects varied, making it challenging to generalize climate effects on lake CHL. For example, all tested climate metrics were related to at least some climate-causal lakes, and 55% were related to more than one climate metric (*SI Appendix*, Fig. S1 and Table S1). Precipitation was related to the largest number of climate-causal lakes (54%), followed by temperature (48%), ENSO (40%), and drought index (38%). For the linear-stochastic models that included the detection of directional effects and time lags, we found little evidence for a consistent effect of climate metrics on lake CHL dynamics (*SI Appendix*, Table S1). Similarly, we did not observe a consistent effect of temperature, which has direct positive relationships to the physiological processes of phytoplankton, particularly some bloom-forming taxa (32–34). Our results are consistent with previous research that found temperature (35, 36), precipitation (37–41), and climate indices such as North American Oscillation and ENSO (42–44) to be related to algal characteristics; however, we document variation in these relationships and report a lack of patterns that apply broadly and consistently.

### Temporal Patterns in 24,452 Lakes.

**Fig. 3.**
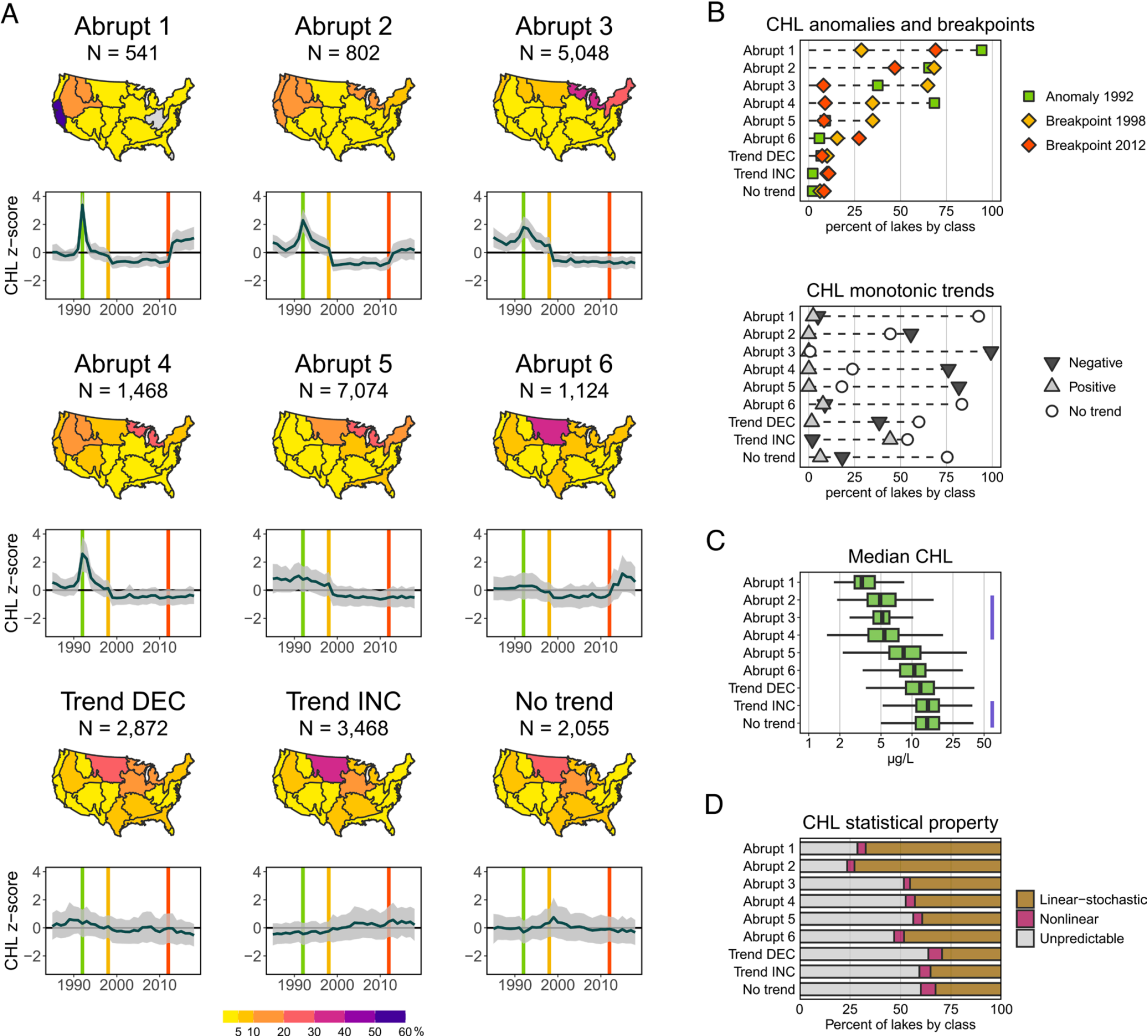
Ecological temporal classes for lake CHL. (*A*) Maps of the percentage of lakes in each NEON region that contain lakes from each ecological temporal class. “Abrupt” classes are those that had lakes with abrupt ecological changes measured by breakpoints and anomalies; “Trend DEC” indicates lakes with decreasing trends; “Trend INC” indicates lakes with increasing trends; “No trend” indicates lakes with no temporal pattern. N is the number of lakes in each class. For each ecological temporal class, plots show the mean lake CHL z-scores (dark gray line) and 1 SD from the mean (gray shading). Colored vertical lines indicate the years with the most lakes demonstrating breakpoints or anomalies. (*B*) Percentage of lake CHL time series by ecological temporal class that showed anomalies or breakpoints for 3 y (*Top*) or monotonic or no trends (*Bottom*). (*C*) Lake long-term median CHL (median of annual means across 34 y) within each class; purple vertical lines indicate statistically similar classes based on pairwise post hoc testing. (*D*) The percentage of lakes with the three different statistical properties by class as defined in Fig. 2.

We provide empirical evidence for the prevalence of nine distinct temporal patterns (i.e., ecological temporal classes; Fig. 3*A*) of lake CHL across thousands of lakes that include well-described patterns from theory and experiments, such as abrupt ecological changes (rapid relative to typical rates of change and can be temporary or persistent) and monotonic trends [directional over time but not always linear (21, 22, 24). The most common patterns (66% of all lakes) are abrupt ecological changes (Abrupt 1 to 6). There was also a class with decreasing monotonic trends (12% of lakes; Trend DEC), a class with increasing monotonic trends (14% of lakes; Trend INC), and a class with no monotonic trends or abrupt changes (8% of lakes; No trend) (Fig. 3*B*).

The lake temporal dynamics within the six abrupt ecological change classes made ecological sense. For example, the highest number of breakpoints or anomalies in CHL occurred in 1992, 1998, and 2012 (Fig. 3*B*). Strong ENSO activity was observed in 1992 and 1998, with low global temperatures in 1992 due to the eruption of Mt. Pinatubo (45), while, in 2012, many regions experienced above-average air temperatures and relatively low precipitation (*SI Appendix*, Fig. S2*A*). The coincident signals in CHL across the conterminous US in these 3 y suggest the influence of broad-scale climate drivers, which appear to result from the common gradual monotonic temperature increases in many regions (*SI Appendix*, Fig. S2*B*), combined with some anomalous decreases (1992) or increases (2012) in temperature for some lake classes (*SI Appendix*, Fig. S2 *A* and *B*).

Our study provides empirical evidence that, of the hundreds to thousands of lakes within an ecological temporal class, any of the time-series statistical properties related to predictability or linearity are possible (Fig. 3*D*). These statistical properties determine our ability to quantify driver–response relationships and indicate the potential for regime shifts. Although all properties are represented in each temporal class, their relative proportions vary in ecologically relevant ways. First, the ecological temporal class assignment was related to long-term median CHL, with nonabrupt lake classes being more likely to have the highest CHL (Fig. 3*C*). High CHL is strongly controlled by human disturbance, such as nutrient inputs from agricultural and urban land uses (45, 46), and human disturbance is believed to increase the likelihood of regime shifts (47). However, compared to the abrupt ecological change classes, the nonabrupt classes with high CHL were only slightly more likely to have nonlinear CHL time series, although they were more likely to be unpredictable (Fig. 3*D*). This result suggests that human-disturbed lakes may be more stochastic or subject to changing human-mediated disturbances through time, limiting our capacity to model their dynamics. Given the small percentage of time series in our study that exhibit the nonlinear properties required for regime shifts, our results suggest that studies of climate effects on lakes should not focus solely on regime shifts, but instead must also include linear-stochastic dynamics and driver–response relationships.

The nine ecological temporal classes were also strongly related to lake environmental context, providing another dimension to lake CHL responses. Decades of research have demonstrated the role of environmental context on average lake CHL, including lake morphometry, watershed soils, hydrology, average climate, and location. Fewer studies have considered these same effects on temporal patterns of lake CHL across many lakes (48). However, we found that many of these characteristics were also related to lake CHL temporal patterns (*SI Appendix*, Figs. S3 and S4). The abrupt lake classes were at the extreme or low ends of ranges for natural and human environmental characteristics, associated with high elevation, cold summer temperatures, low human land use, and extreme hydrology (characterized by high groundwater recharge and coarse soils). These results are supported by previous studies documenting the responsiveness of mountain (49) and high-latitude (50) lakes to global change, as well as the responsiveness of lakes with low human disturbance to climate change (51). Here, we document these patterns across thousands of lakes and compare them to lakes with contrasting characteristics, particularly highly disturbed lakes.

### Climate-Causal Relationships to Environmental Context and Ecological Temporal Class.

**Fig. 4.**
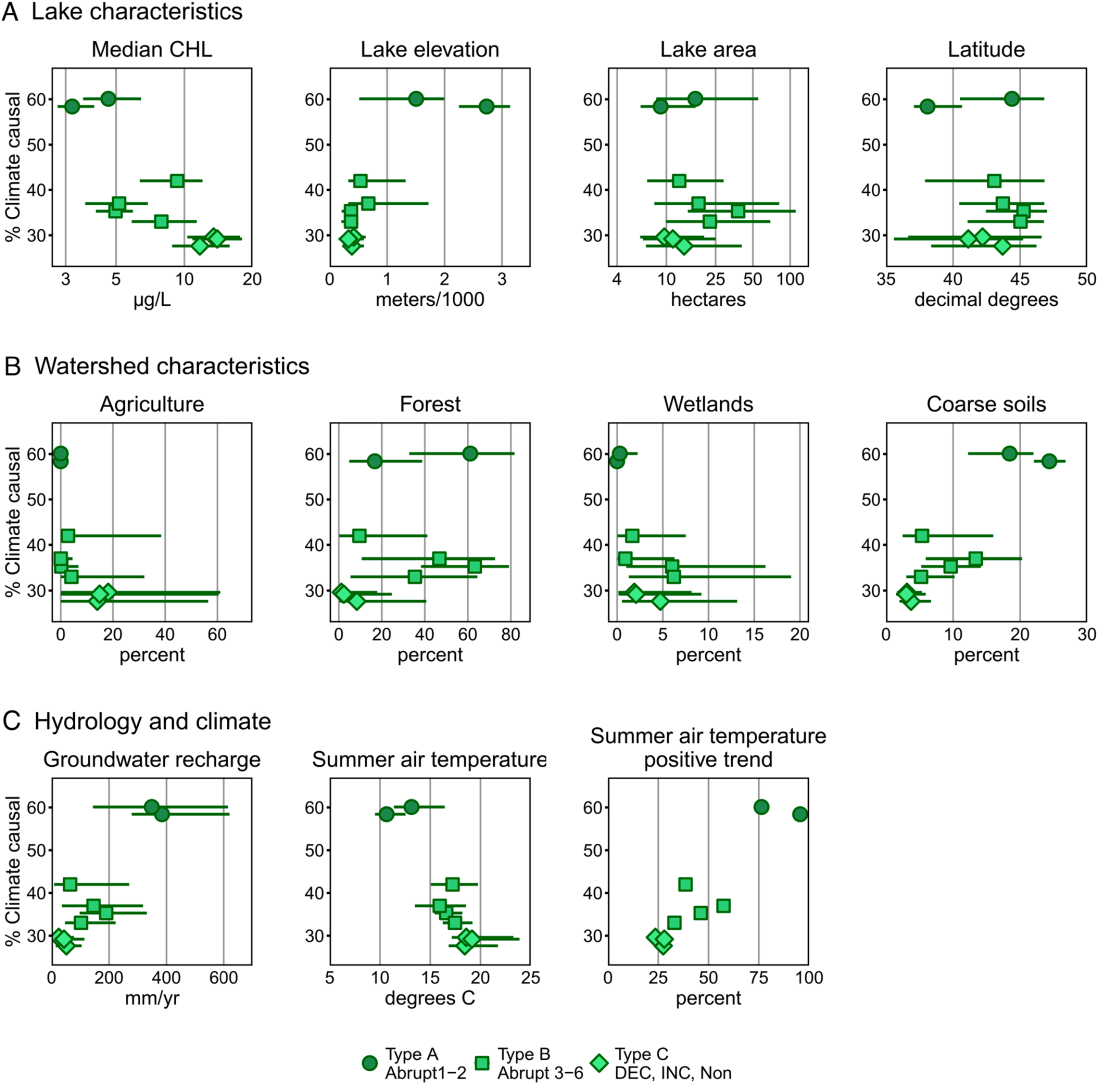
Relationships between the % climate-causal lakes in ecological temporal classes and environmental context characteristics grouped into climate-response types. (*A*) Lake characteristics (long-term median CHL, lake elevation, lake area, and latitude). (*B*) Watershed characteristics (% agriculture, % forest, % wetlands, and % coarse soils). (*C*) Hydrology and climate (groundwater recharge, median summer air temperature, and % of lakes with a positive trend in summer air temperature). Data are for climate-causal lakes within each climate-response type. Lines indicate the interquartile range; long-term median CHL and lake area are plotted on a log scale. Ecological temporal class labels are as for Fig. 3. The *Y* axis in all plots begins at 25%.

We found that temporal pattern, defined by ecological temporal class, and temporal process, defined by climate-causality, were related and helped to explain climate-response types across thousands of lakes (Fig. 4). The highest percentage of lakes that were causally related to climate (33 to 60%) were in ecological temporal classes with abrupt changes (Type A and B). Whereas the lowest percentages of lakes related to climate (28 to 30%) were in ecological temporal classes with either increasing or decreasing monotonic trends or no temporal patterns (Type C). Further, the abrupt classes that were at the extremes of many natural environmental ranges had the largest percentage of lakes causally related to climate, a result that supports the expectation that this class of lakes is most likely to have responded to climate to date (49, 50). However, we add context to this expectation by including lakes with wide ranges of human disturbance in our study. Lakes with moderate to high CHL and significant human disturbances were least likely to respond to climate. These lakes tended to show monotonic trends or no patterns, rather than abrupt ecological changes, supporting the idea that human disturbances may mask the abrupt CHL responses to climate that are observed in low-disturbance lakes. Although many of these environmental characteristics covary (such as lake elevation, median summer temperature, % agriculture, and % coarse soils), these factors likely covary across the globe and provide insight into where lakes may be most responsive to climate and how—such as through abrupt ecological changes, rather than smooth linear changes. In addition, based on the relationship with the positive trend in summer air temperature, it is possible that if the temperature trends of the 34-y study period become more widespread or intensify, a larger proportion of lakes may exhibit climate-causal responses—although the nature of the response is also mediated by ecological context and human disturbance.

### A Framework for Climate Effects on Ecosystems at Macroscales.

**Fig. 5.**
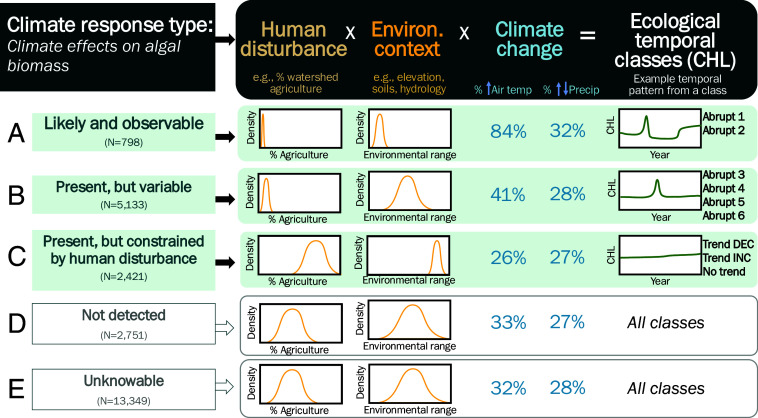
A framework for understanding climate effects on ecosystems at the continental-scale, with lake CHL as a model. By integrating temporal process (i.e., climate causality) and pattern (ecological temporal class), it is possible to identify the environmental contexts and temporal dynamics likely to be indicative of climate causing the dynamics in CHL. Based on our documented climate effects on lake CHL, we propose five climate response types defined by human disturbance, environmental context, and climate change that are reflected in a lake’s ecological temporal pattern. Three combinations of these factors (Types A-C) represent distinct climate responses that we quantified for the study lakes. Type D lake time series were predictable but were not related to any climate metric; Type E lake time series were not predictable and could not be modeled. The human disturbance and environmental context graphs depict density plots of a single characteristic for all lakes within a type to indicate narrow or broad ranges of characteristics.

Climate effects on ecosystems at macroscales are heterogeneous and can be understood as a function of human disturbance, environmental context, and recent changes in air temperature and precipitation that are all reflected in the observed ecological temporal patterns. We provide a framework (Fig. 5) that describes the conditions under which climate-causal relationships with an ecosystem response, such as algal biomass, are likely to occur. This framework can be used as a conceptual model for predicting future responses and temporal patterns across broader ranges of climate and environmental contexts or for other ecosystem types and responses. We demonstrate that process by describing how this framework applies to lake algal biomass response to climate (Fig. 5).

In our study of thousands of lakes across diverse environmental settings, we found three types of responses that describe climate effects on CHL: *likely and observable* (Type A—Abrupt 1 and 2); *present, but variable* (Type B—Abrupt 3 to 6); and *present but constrained by human disturbance* (Type C—Nonabrupt). Type A lakes were least affected by humans, had the lowest long-term median CHL levels, and were at environmental extremes of low summer temperatures and high elevations. These lakes exhibit strong, potentially predictable response patterns to climate, have large and frequent abrupt ecological changes, as measured by breakpoints and anomalies, and have experienced some of the strongest changes in climate in the 34-y study period (mainly in air temperature, with a significant increase in air temperature observed in 84% of these lakes). Studies support this result in which lakes in extreme environments are responsive to air temperature (49, 50) or the interaction between air temperature and the amount of precipitation as snow in high-elevation lakes (52).

Type C lakes were the least likely to be causally related to climate and had higher levels of human disturbance and long-term median CHL. Type C lakes were located at opposite environmental extremes to Type A, with high algal biomass likely caused by high nutrient loads from agriculture (*SI Appendix*, Fig. S9). In these lakes, CHL temporal dynamics were more muted, with far fewer abrupt changes than observed in Type A or B lakes despite 26% and 27% of the lakes experiencing increased air temperatures or changing precipitation, respectively. We speculate that CHL in Type C lakes is more directly driven by land–water interactions that control external nutrient loads, which are sensitive to precipitation extremes (18, 53–55). Therefore, the climate signals recorded in the 34-y study period may have been more indirect and overshadowed by the larger effects of human disturbance, leading to a somewhat muted direct effect of climate on CHL in this climate-response type. Given the ongoing intensification of weather extremes and reconfiguring of precipitation into more intense storms and droughts, we predict Type C lakes will have stronger responses to climate change in the future, particularly if warming temperatures are combined with major changes in precipitation (40, 56–58).

Type B lakes fell between these two extremes, with climate causing changes in CHL, but to varying degrees for the different classes of lakes within the Type. The lakes had intermediate environmental context ranges, low to moderate levels of human disturbance, and low to moderate long-term median CHL levels; 41% of this class of lakes also experienced increased air temperatures. Although these lakes have abrupt temporal patterns, their moderate climate responsiveness highlights the complex, multiscale interactions between external and internal factors that control lake CHL.

The remaining two types of lake CHL time series were unrelated to climate. Type D lakes had predictable CHL time series, but no significant effects of climate were present. Type D lakes were distributed across all lake ecological temporal classes and did not stand out as having any differentiating environmental context characteristics (*SI Appendix*, Fig. S9). We speculate that, as the time series record lengthens and climate change intensifies, we may be able to estimate more Type D relationships that do not currently reach significance, perhaps due to a lack of statistical power. Finally, Type E lakes have stochastic CHL time series that remain unpredictable limiting our ability to estimate climate causality; it remains to be seen whether the number of unknowable relationships of this type will change with longer observation periods and continued climate change. Interestingly, these two types of climate-response lakes do not appear to have specific characteristics; they span a broad range of environmental characteristics, as well as ecological temporal patterns (*SI Appendix*, Fig. S9).

Our study provides empirical evidence that algal biomass in one-third of 24,452 lakes across diverse ecoclimatic zones responded to changing climate patterns from 1985 to 2018. Although temperature increased in many regions, these increases did not appear to lead to widespread and consistent increases in algal biomass. Additionally, although regime shifts are possible, they appear rare at the temporal scales and under the climate conditions we studied. Instead, algal biomass responded to climate across a broad range of lakes—including those with both low and high levels of human disturbance and diverse CHL temporal patterns. However, climate causality was more likely in lakes with low CHL, minimal human disturbances, extreme environmental conditions, and abrupt ecological changes than in lakes without those characteristics. In contrast, lakes with moderate to high CHL and significant human disturbances were less likely to respond to climate. These lakes tended to show monotonic trends or no patterns rather than abrupt ecological changes, supporting the idea that human disturbances may mask the abrupt CHL responses to climate observed in low-disturbance lakes. Finally, over half of the tested lakes had 34-y time series that were too unpredictable to model. Therefore, it is important to continue testing for climate effects as time series records lengthen so we can improve our ability to detect effects in an even broader range of lakes.

Reconciling hypotheses about how lake algal biomass will respond to intensifying climate change, increasing climate variability, and escalating human activities over the coming decades is an urgent priority. To address this need, we developed a framework that can be applied to other ecosystem types and regions worldwide. This framework captures the heterogeneous responses of ecosystem properties to the joint effects of climate, human disturbance, and local environmental conditions. Because of these complex interactions, climate effects cannot be considered separately from the wide range of human disturbances and environmental conditions existing across the globe, and scientists should anticipate a diversity of responses. Our approach demonstrates the value of integrating temporal pattern and process to understand and generalize ecosystem responses to climate at continental to global scales.

## Materials and Methods

### Study Area and Lakes.

The conterminous US includes abundant lakes and broad climate ranges located in four of the seven world biomes: temperate forest, desert, savannah, and grassland which were delineated into regions using NEON domains (59). Our study scope included all 137,465 lakes ≥4 ha, excluding the Great Lakes (60). We excluded lakes <4 ha because of the limitations in extracting information from Landsat imagery on very small lakes (see below). 24,452 of these lakes had sufficient data coverage for time series analysis with nearly complete 34-y CHL records from 1985 to 2018 and complete lake and watershed ecological context characteristics.

### Data.

#### Algal biomass data.

The LAGOS-US LANDSAT module provided lake CHL (a measure of algal biomass) for lakes ≥4 ha derived from Landsat satellite imagery (extensive methodology, QAQC, and validation for the Landsat-derived lake CHL are provided in refs. 25 and 26). Briefly, all median surface reflectance bands and pixel-specific band ratios for water pixels were used from the Level-2 Surface Reflectance Product after band harmonization across Landsat sensors following Roy et al. (61, 62). Scenes were excluded when cloud cover was ≥50%, if a retrieval had <10% of the maximum water pixel coverage from a lake’s overpasses, or if any band’s median value was negative. Retrievals were matched to in situ CHL from LAGOS-US LIMNO for up to a 7-d window. A random forest regression was built on all bands and band ratios for ±1-d matchups to predict CHL for all lake and Landsat scene combinations. The model predictions for median summer CHL had a mean absolute percentage error of 25.3% CHL and an R^2^ of 0.52 (*P* < 0.0001).

Median summer values for CHL for each lake and year were calculated from ≥eight April–October CHL values with no two consecutive Landsat overpasses (16-d return interval) excluded. Lakes were allowed to have five missing nonconsecutive years of data. The final dataset included 24,452 lakes in which 46.1% had complete time series, 27.1% were missing a single year, 14.7% were missing 2 y, 7.8% were missing 3 y, 4.2% were missing 4 y, and only 0.01% were missing 5 y. We infilled missing single years of data with the mean of the previous and subsequent years. Missing first or last years were infilled with the one adjacent year’s value.

The final study population of lakes with nearly complete CHL time series is distributed across the conterminous US (*SI Appendix*, Fig. S5) and is representative of lakes within regions, based on important ecological context variables, which demonstrates that the study population is not biased (*SI Appendix*, Figs. S6 and S7). However, there is spatial patterning in the final lake population (*SI Appendix*, Fig. S5) most likely due to two issues that arise when building consecutive time series from satellite data. First, spatial patterning can result from areas of the country that are more likely to have summer cloud cover which limits the ability to detect lake CHL. Second, sufficiently clear Landsat scenes are more likely where the Landsat scenes had row/path combinations that overlap, leading to the striped patterning. Despite these constraints, the lakes in the final population covered all regions of the United States (*SI Appendix*, Table S2) and there were no obvious biases in ecological settings for the lakes in the final dataset (*SI Appendix*, Figs. S6 and S7).

#### Climate data.

We classified climate variables into four categories of climate metrics (ENSO, drought, precipitation, and air temperature). Monthly, downscaled values for mean air temperature (hereafter, temperature) and total precipitation were obtained from PRISM at 4 km grid cell resolution (63) and compiled for lakes at the HU12 spatial scale (64). Drought was characterized by the monthly Palmer Hydrological Drought Index (NOAA). For the ENSO metric, we used the bimonthly multivariate ENSO index (MEI.v2), which is standardized to the 1980-2018 reference period (NOAA). We compiled annual values for the four climate metrics at four different intra-annual temporal scales (16 total): water year (preceding year October to focal year September), spring (February–April), summer (May–September), and preceding winter (November–January) (39).

#### Environmental context data.

The LAGOS-US Research Platform (60) was used to characterize lakes by location and physical characteristics of lakes and their watersheds using the LOCUS module (65), and by ecological context using the GEO module (64). For each lake, we quantified characteristics in seven major categories that are known to influence algal biomass: climate normals, lake location, lake and watershed morphometry, watershed terrain, watershed soils, watershed land use/cover, and hydrology.

### Methods Overview.

We integrated the rich Landsat library—now sufficient for time series modeling—with extensive fine-scale geospatial data products. We estimated temporal process (climate causality) using two types of time series models suitable for either linear-stochastic or nonlinear time series. Additionally, we estimated temporal patterns using a machine-learning clustering algorithm and statistical pattern analyses. We had three main components to our analysis, including two major analytical streams focused on either pattern or process and a synthesis of the two (Fig. 1). First, to quantify the causal relationship between climate and lake CHL time series, we used time series models of each of the lake CHL time series (i.e., process). Second, to help understand the temporal ecology of the lake CHL time series, we conducted a temporal pattern analysis of the lake CHL time series (i.e., pattern). Third, to understand how environmental context influences the climate effects on algal biomass, we synthesized the results from both approaches to integrate temporal pattern and process. All code (66) and data (see above) are available for download.

### Process: Time Series Modeling.

Time series were analyzed using empirical dynamic modeling using the rEDM R package (61) following Clark and Luis (27) that included first estimating whether the time series were predictable. Nonpredictable time series were excluded from further analyses. Based on the statistical properties of a predictable lake’s CHL time series dynamics, we used one of two time series model approaches to quantify the causality of climate on CHL.

#### Statistical dynamics.

Preprocessing included scaling, standardizing, and detrending each lake’s individual CHL time series. Time series predictability was tested using out-of-sample “forecast skill” based on S-maps (27). Time series were classified as predictable if the Pearson correlation coefficient of leave-one-out cross-validation of the actual and predicted time series was statistically significant (*P* < 0.05). S-maps determine predictability by measuring the divergence of the estimated probability density function from the true probability density function. We estimated the embedding dimension (E, the number of time lags needed to reconstruct the state-space using lagged coordinates) for each lake CHL time series using simplex projection and classified each lake CHL time series as nonlinear or linear-stochastic using sequential locally weighted global linear maps (S-maps). Like Clark and Luis (27), we varied E from 1 to 10 for simplex projections, identified the best E for each time series, applied that to the S-maps, and varied the nonlinear tuning parameter from 0 to 8. To determine whether an individual lake CHL time series was nonlinear or linear-stochastic, we used a randomization procedure to estimate *P*-values to determine whether the change in mean absolute error (ΔMAE) from a linear to nonlinear model was positive and significant (*P* < 0.05). The ΔMAE was estimated by subtracting the minimum MAE from the global linear model MAE. The *P*-value was estimated by generating 1,000 phase-randomized time series using the surrogates function from the astrochron R library (67) that preserved the statistical properties of the original time series (e.g., autocorrelation) and introduced random noise. This process created a null distribution of ΔMAE to compare the ΔMAE estimated from the actual time series.

#### Causality for nonlinear time series.

For nonlinear CHL time series that were predictable, we determined to what degree any of the 16 climate-by-season variables were driving the lake CHL time series using convergent cross mapping, which infers a causal relationship between a driver variable (e.g., a climate metric) and a response variable (e.g., the individual lake CHL time series). We used the rEDM R package following Liu and Gaines (67). We performed convergent cross mapping for each lake CHL time series and climate variable combination using the best E identified above. Two criteria were used to infer causality and minimize capturing spurious correlations (67, 68). First, we ensured that the cross-map skill using all data was significantly greater than zero. Second, we confirmed that predictability was convergent by ensuring that the predictive skill at the maximum library size (library size = 500) was greater than the predictive skill at the minimum library size [library size = 20; (67, 69)]. When both criteria were met, a climate variable was inferred to have a causal relationship with lake CHL.

#### Causality for linear-stochastic time series.

For predictable linear-stochastic CHL time series, we determined to what degree any of the 16 climate-by-season variables were driving the lake CHL time series using Granger and instantaneous causality, which tests whether the prediction of a time series improves using the information contained within a second-time series (70). We fit vector autoregression models to each lake CHL time series for each climate driver with a lag of 1 y. A climate driver can be found to be either independent of CHL, Granger-causal only, instantaneous-causal only, or both Granger- and instantaneous-causal. Granger causality employs a lag between driver and effect, whereas instantaneous causality does not rely on a lag and provides a less structured causal link (71). The time series models were tested for Granger and instantaneous causality using the “lmtest” R package (72). Climate drivers were considered Granger- or instantaneous-causal if the *P* < 0.05 for the respective test.

We set our statistical threshold for all analyses at *P* < 0.05 for the four different analytical steps of our analysis that have complex estimated parameters for each lake. We chose not to select a lower *P* value threshold to maximize our power to detect effects due to the relatively short time series (in the absence of knowing the power sensitivity at each analysis step). We accept that this decision increases the potential for Type I errors.

### Pattern: Temporal Pattern Analysis and Environmental Context.

#### Hierarchical clustering.

We converted each lake’s time series to a z-score (mean = 0, SD = 1) to prioritize temporal patterns and control for CHL magnitude. CHL time series were clustered using agglomerative hierarchical clustering using scikit-learn in Python (73) by initially treating each lake as its own cluster, then recursively merging clusters step-wise while subject to a criterion (30, 74, 75). We used Ward’s method as our criterion, which merges clusters that minimize within-cluster variance (76), which achieves our objective of clustering lakes with similar interannual temporal patterns of CHL. We selected the 16-cluster output that balanced cluster granularity and generality. We further group these clusters into ecological temporal classes as described below.

#### Calculation of temporal pattern indicators using breakpoints, anomalies, and monotonic trends.

To quantitatively describe the cluster CHL temporal patterns, we analyzed each lake’s CHL time series for breakpoints, anomalies, and 34-y monotonic trend. Breakpoints were calculated using the strucchange R package (77) with a minimum segment size of 15% of the CHL time series length and using a method that implements multiple, simultaneous breakpoint determinations (78). A year was defined as a breakpoint if the CHL time series shifted from one stable linear regression relationship to another in that year. A year was defined as having an anomaly if the within-lake CHL z-score for that year was beyond ±1 SD. We identified each lake’s monotonic 34-y CHL trend using a Mann-Kendall test based on a two-sided *P* < 0.05 and directionality based on the sign of Kendall’s τ.

#### Creating ecological temporal classes.

We analyzed the existing 16 clusters to determine the classes that could be combined that had similar temporal pattern indicators (breakpoints, anomalies, or monotonic trends and directions), but perhaps simply different magnitudes suggesting similar ecological drivers. By evaluating these pattern indicators, we found that the 16 clusters could be reduced to nine ecological temporal classes, defined as classes with similar ecological temporal patterns. We first identified six possible classes if one ignores the year of breakpoint or anomaly occurrences: 1) abrupt with temporary change (time series ≥ one anomalous year); 2) abrupt with persistent change (time series with ≥ one breakpoint); 3) abrupt with temporary and persistent change (time series with ≥ one breakpoint and ≥ one anomaly); 4) monotonic-increasing (time series with no anomalies or breakpoints, but with increasing trend); 5) monotonic-decreasing (time series with no anomalies or breakpoints, but with decreasing trend); 6) no pattern (no detected breakpoints or anomalies). We then split out classes by documenting the year of the anomalies and breakpoints to create a final ecological temporal classification that contained nine classes. For example, if there were three lake clusters with abrupt but temporary changes that occurred in different years, those lakes were assigned to different classes as they may indicate responses to different drivers. In contrast, if two clusters had abrupt but persistent changes in the same year, but one cluster had larger changes than the others, they were grouped into the same class. Of the nine ecological classes, six contained any kind of abrupt change (Abrupt 1 to 6), one class had decreasing monotonic trends (Trend DEC), one class with increasing monotonic trends (Trend INC), and one class with no quantifiable patterns (No trend). We validated these ecological temporal classes with two approaches. First, we plotted a broad range of ecological context characteristics (including lake, watershed, terrain, soils, land use, and hydrology) across the classes and found strong patterns by class (*SI Appendix*, Fig. S3). Second, we modeled the effect of 32 watershed and lake characteristics on the refined class assignments using a GLMnet model which supported a strong association between class assignment and many of the environmental context variables (e.g., elevation, latitude, lake area, soils, and land use).

### Integration: Climate-Causal Relationships to Environmental Context and Temporal Class.

Using the ecological temporal classification, we calculated the proportion of lake CHL time series in each class that were causally related to any climate metric. We then plotted these against ecological context characteristics to identify those features that may be most strongly related to climate causality of lake algal biomass. To examine how the temporal patterns (i.e., the ecological temporal class assignment) were related to process (i.e., climate causality), we examined which ecological context characteristics had the strongest relationships to document the environmental conditions most likely to lead to algal biomass responses to climate. As a final synthesis, we identified five possible climate response types for algal biomass that included all 24,452 lakes in our study population.

## Supplementary Material

Appendix 01 (PDF)

## Data Availability

The original data used for this article were downloaded from https://portal.edirepository.org/nis/mapbrowse?packageid=edi.1427.1 (25); the processed and filtered dataset (tables of lake productivity, environmental data, and model output) is available at https://doi.org/10.5281/zenodo.10926306 (79). Code can be found at https://doi.org/10.5066/P15PMPVG (66).

## References

[1] J. S. Hsu, J. Powell, P. B. Adler, Sensitivity of mean annual primary production to precipitation. Glob. Change Biol. **18**, 2246–2255 (2012).

[2] G. E. Maurer, A. J. Hallmark, R. F. Brown, O. E. Sala, S. L. Collins, Sensitivity of primary production to precipitation across the United States. Ecol. Lett. **23**, 527–536 (2020). 31912647 10.1111/ele.13455

[3] IPCC, “Climate change 2022: Impacts, adaptation, and vulnerability” in Contribution of Working Group II to the Sixth Assessment Report of the Intergovernmental Panel on Climate Change, H.-O. Pörtner , Eds. (Cambridge University Press, 2022).

[4] T. Sasaki , Dryland sensitivity to climate change and variability using nonlinear dynamics. Proc. Natl. Acad. Sci. U.S.A. **120**, e2305050120 (2023). 37603760 10.1073/pnas.2305050120 PMC10587894

[5] G. Kulk , Correction: Kulk et al. Primary production, an index of climate change in the ocean: Satellite-based estimates over two decades. Remote Sens. 2020, 12, 826. Remote Sens. **13**, 3462 (2021).

[6] L. J. Gilarranz, A. Narwani, D. Odermatt, R. Siber, V. Dakos, Regime shifts, trends, and variability of lake productivity at a global scale. Proc. Natl. Acad. Sci. U.S.A. **119**, e2116413119 (2022). 35994657 10.1073/pnas.2116413119 PMC9436327

[7] M. Scheffer, S. R. Carpenter, Catastrophic regime shifts in ecosystems: Linking theory to observation. Trends Ecol. Evol. **18**, 648–656 (2003).

[8] A. M. Michalak, Study role of climate change in extreme threats to water quality. Nature **535**, 349–350 (2016). 27443725 10.1038/535349a

[9] M. Ryo, C. A. Aguilar-Trigueros, L. Pinek, L. A. H. Muller, M. C. Rillig, Basic principles of temporal dynamics. Trends Ecol. Evol. **34**, 723–733 (2019). 31010706 10.1016/j.tree.2019.03.007

[10] C. Hsieh, S. M. Glaser, A. J. Lucas, G. Sugihara, Distinguishing random environmental fluctuations from ecological catastrophes for the North Pacific Ocean. Nature **435**, 336–340 (2005). 15902256 10.1038/nature03553

[11] S. Sadro, C. E. Nelson, J. M. Melack, The influence of landscape position and catchment characteristics on aquatic biogeochemistry in high-elevation lake-chains. Ecosystems **15**, 363–386 (2012).

[12] N. M. Hayes, M. J. Vanni, M. J. Horgan, W. H. Renwick, Climate and land use interactively affect lake phytoplankton nutrient limitation status. Ecology **96**, 392–402 (2015). 26240861 10.1890/13-1840.1

[13] B. M. Kraemer, T. Mehner, R. Adrian, Reconciling the opposing effects of warming on phytoplankton biomass in 188 large lakes. Sci. Rep. **7**, 1–7 (2017). 28883487 10.1038/s41598-017-11167-3 PMC5589843

[14] T. C. Ballard, E. Sinha, A. M. Michalak, Long-term changes in precipitation and temperature have already impacted nitrogen loading. Environ. Sci. Technol. **53**, 5080–5090 (2019). 30979339 10.1021/acs.est.8b06898

[15] A. R. Hrycik , Earlier winter/spring runoff and snowmelt during warmer winters lead to lower summer chlorophyll-*a* in north temperate lakes. Glob. Change Biol. **27**, 4615–4629 (2021). 10.1111/gcb.15797 34241940

[16] A. Paltsev, I. F. Creed, Multi-decadal changes in phytoplankton biomass in northern temperate lakes as seen through the prism of landscape properties. Glob. Change Biol. **28**, 2272–2285 (2022). 10.1111/gcb.16079 35014108

[17] E. Jeppesen , Climate change effects on runoff, catchment phosphorus loading and lake ecological state, and potential adaptations. J. Environ. Qual. **38**, 1930–1941 (2009). 19704137 10.2134/jeq2008.0113

[18] R. L. G. Nobre , Precipitation, landscape properties and land use interactively affect water quality of tropical freshwaters. Sci. Total Environ. **716**, 137044 (2020). 32059302 10.1016/j.scitotenv.2020.137044

[19] K. Song , Climatic versus anthropogenic controls of decadal trends (1983–2017) in algal blooms in lakes and reservoirs across China. Environ. Sci. Technol. **55**, 2929–2938 (2021). 33595308 10.1021/acs.est.0c06480

[20] M. Scheffer, Critical Transitions in Nature and Society (Princeton University Press, 2009).

[21] M. G. Turner , Climate change, ecosystems and abrupt change: Science priorities. Philos. Trans. R. Soc. B Biol. Sci. **375**, 20190105 (2020). 10.1098/rstb.2019.0105 PMC7017767 31983326

[22] J. W. Williams, J. L. Blois, B. N. Shuman, Extrinsic and intrinsic forcing of abrupt ecological change: Case studies from the late quaternary. J. Ecol. **99**, 664–677 (2011).

[23] E. M. Wolkovich, B. I. Cook, K. K. McLauchlan, T. J. Davies, Temporal ecology in the Anthropocene. Ecol. Lett. **17**, 1365–1379 (2014). 25199649 10.1111/ele.12353

[24] Z. Ratajczak , Abrupt change in ecological systems: Inference and diagnosis. Trends Ecol. Evol. **33**, 513–526 (2018). 29784428 10.1016/j.tree.2018.04.013

[25] P. J. Hanly, K. E. Webster, P. A. Soranno, LAGOS-US LANDSAT: Data module of remotely-sensed water quality estimates for U.S. lakes over 4 ha from 1984 to 2020. Environmental Data Initiative (2024). https://portal-s.edirepository.org/nis/mapbrowse?scope=edi&identifier=1427 & revision=2. 10.1038/s41597-025-05600-w PMC12307578 40730551

[26] P. J. Hanly, K. E. Webster, P. A. Soranno, LAGOS-US LANDSAT: Remotely sensed water quality estimates for U.S. lakes over 4 ha from 1984 to 2020. bioRxiv [Preprint] (2024). 10.1101/2024.05.10.593626 (Accessed 30 October 2024). PMC12307578 40730551

[27] T. J. Clark, A. D. Luis, Nonlinear population dynamics are ubiquitous in animals. Nat. Ecol. Evol. **4**, 75–81 (2020). 31819235 10.1038/s41559-019-1052-6

[28] G. Sugihara , Detecting causality in complex ecosystems. Science **338**, 496–500 (2012). 22997134 10.1126/science.1227079

[29] M. C. McGraw, E. A. Barnes, Memory matters: A case for granger causality in climate variability studies. J. Clim. **31**, 3289–3300 (2018).

[30] F. Murtagh, P. Contreras, Algorithms for hierarchical clustering: An overview, II. WIREs Data Min. Knowl. Discov. **7**, e1219 (2017).

[31] M. G. Turner, R. H. Gardner, Landscape Ecology in Theory and Practice: Pattern and Process (Springer, 2015).

[32] H. W. Paerl, J. Huisman, Blooms like it hot. Science **320**, 57–58 (2008). 18388279 10.1126/science.1155398

[33] R. Adrian , Lakes as sentinels of climate change. Limnol. Oceanogr. **54**, 2283–2297 (2009). 20396409 10.4319/lo.2009.54.6_part_2.2283 PMC2854826

[34] R. I. Woolway, S. Sharma, J. P. Smol, Lakes in hot water: The impacts of a changing climate on aquatic ecosystems. BioScience **72**, 1050–1061 (2022). 36325103 10.1093/biosci/biac052 PMC9618276

[35] R. D. Robarts, T. Zohary, Temperature effects on photosynthetic capacity, respiration, and growth rates of bloom-forming cyanobacteria. N. Z. J. Mar. Freshw. Res. **21**, 391–399 (1987).

[36] J. E. Bissinger, D. J. S. Montagnes, J. Harples, D. Atkinson, Predicting marine phytoplankton maximum growth rates from temperature: Improving on the Eppley curve using quantile regression. Limnol. Oceanogr. **53**, 487–493 (2008).

[37] K. C. Rose, S. R. Greb, M. Diebel, M. G. Turner, Annual precipitation regulates spatial and temporal drivers of lake water clarity. Ecol. Appl. **27**, 632–643 (2017). 27859882 10.1002/eap.1471

[38] J. Richardson , Response of cyanobacteria and phytoplankton abundance to warming, extreme rainfall events and nutrient enrichment. Glob. Change Biol. **25**, 3365–3380 (2019). 10.1111/gcb.14701 PMC6852574 31095834

[39] S. M. Collins , Winter precipitation and summer temperature predict lake water quality at macroscales. Water Resour. Res. **55**, 2708–2721 (2019).

[40] J. C. Ho, A. M. Michalak, N. Pahlevan, Widespread global increase in intense lake phytoplankton blooms since the 1980s. Nature **574**, 667–670 (2019). 31610543 10.1038/s41586-019-1648-7

[41] P. D. F. Isles , Widespread synchrony in phosphorus concentrations in northern lakes linked to winter temperature and summer precipitation. Limnol. Oceanogr. Lett. **8**, 639–648 (2023).

[42] D. Straile, R. Adrian, The North Atlantic Oscillation and plankton dynamics in two European lakes—Two variations on a general theme. Glob. Change Biol. **6**, 663–670 (2000).

[43] T. Blenckner , Large-scale climatic signatures in lakes across Europe: A meta-analysis. Glob. Change Biol. **13**, 1314–1326 (2007).

[44] X. Xiao , Teleconnection between phytoplankton dynamics in north temperate lakes and global climatic oscillation by time-frequency analysis. Water Res. **154**, 267–276 (2019). 30802701 10.1016/j.watres.2019.01.056

[45] S. R. Carpenter , Nonpoint pollution of surface waters with phosphorus and nitrogen. Ecol. Appl. **8**, 559–568 (1998).

[46] L. Moslenko, K. Blagrave, A. Filazzola, A. Shuvo, S. Sharma, Identifying the influence of land cover and human population on chlorophyll a concentrations using a pseudo-watershed analytical framework. Water **12**, 3215 (2020).

[47] J. C. Rocha, G. D. Peterson, R. Biggs, Regime shifts in the Anthropocene: Drivers, risks, and resilience. PLoS One **10**, e0134639 (2015). 26267896 10.1371/journal.pone.0134639 PMC4533971

[48] N. R. Lottig , Macroscale patterns of synchrony identify complex relationships among spatial and temporal ecosystem drivers. Ecosphere **8**, e02024 (2017).

[49] K. A. Moser , Mountain lakes: Eyes on global environmental change. Glob. Planet. Change **178**, 77–95 (2019).

[50] J. E. Saros , Sentinel responses of Arctic freshwater systems to climate: Linkages, evidence, and a roadmap for future research. Arct. Sci. **9**, 356–392 (2023).

[51] K. Kakouei , Phytoplankton and cyanobacteria abundances in mid-21st century lakes depend strongly on future land use and climate projections. Glob. Change Biol. **27**, 6409–6422 (2021). 10.1111/gcb.15866 34465002

[52] S. Sadro, J. M. Melack, J. O. Sickman, K. Skeen, Climate warming response of mountain lakes affected by variations in snow. Limnol. Oceanogr. Lett. **4**, 9–17 (2019).

[53] P. A. Soranno, S. L. Hubler, S. R. Carpenter, R. C. Lathrop, Phosphorus loads to surface waters: A simple model to account for spatial pattern of land use. Ecol. Appl. **6**, 865–878 (1996).

[54] E. Sinha, A. M. Michalak, V. Balaji, Eutrophication will increase during the 21st century as a result of precipitation changes. Science **357**, 405–408 (2017). 28751610 10.1126/science.aan2409

[55] B. Qin , Extreme climate anomalies enhancing cyanobacterial blooms in Eutrophic Lake Taihu, China. Water Resour. Res. **57**, e2020WR029371 (2021).

[56] S. R. Loarie , The velocity of climate change. Nature **462**, 1052–1055 (2009). 20033047 10.1038/nature08649

[57] E. M. Schliep, A. E. Gelfand, J. S. Clark, Stochastic modeling for velocity of climate change. J. Agric. Biol. Environ. Stat. **20**, 323–342 (2015).

[58] K. A. Warner, J. E. Saros, Variable responses of dissolved organic carbon to precipitation events in boreal drinking water lakes. Water Res. **156**, 315–326 (2019). 30927627 10.1016/j.watres.2019.03.036

[59] W. W. Hargrove, F. M. Hoffman, Using multivariate clustering to characterize ecoregion borders. Comput. Sci. Eng. **1**, 18–25 (1999).

[60] K. S. Cheruvelil , LAGOS-US LOCUS v1.0: Data module of location, identifiers, and physical characteristics of lakes and their watersheds in the conterminous U.S. Limnol. Oceanogr. Lett. **6**, 270–292 (2021).

[61] H. Ye, A. Clark, E. Deyle, G. Sugihara, rEDM: An R package for empirical dynamic modeling and convergent cross mapping. Github. https://ha0ye.github.io/rEDM/articles/rEDM.html. Accessed 25 August 2022.

[62] D. P. Roy , Characterization of Landsat-7 to Landsat-8 reflective wavelength and normalized difference vegetation index continuity. Remote Sens. Environ. **185**, 57–70 (2016). 10.1016/j.rse.2015.12.024 PMC6999663 32020954

[63] PRISM Climate Group, LT–Monthly time series (LT81m). http://prism.oregonstate.edu. Accessed 17 January 2019.

[64] N. J. Smith, K. E. Webster, L. K. Rodriguez, K. S. Cheruvelil, P. A. Soranno, LAGOS-US GEO v1.0: Data module of lake geospatial ecological context at multiple spatial and temporal scales in the conterminous U.S. Environmental Data Initiative. 10.6073/PASTA/0E443BD43D7E24C2B6ABC7AF54CA424A. Deposited 18 September 2022.

[65] N. J. Smith, K. E. Webster, L. K. Rodriguez, K. S. Cheruvelil, P. A. Soranno, LAGOS-US LOCUS v1.0: Data module of location, identifiers, and physical characteristics of lakes and their watersheds in the conterminous U.S. Environmental Data Initiative. 10.6073/pasta/e5c2fb8d77467d3f03de4667ac2173ca. Deposited 15 June 2021.

[66] T. Wagner, A. McDonald, P. J. Hanly, R and Python code for analysis of productivity in thousands of US lakes in response to climate over the last 30 years. USGS Information Product Data System. 10.5066/P15PMPVG. Deposited 7 February 2024.

[67] O. R. Liu, S. D. Gaines, Environmental context dependency in species interactions. Proc. Natl. Acad. Sci. U.S.A. **119**, e2118539119 (2022). 36037344 10.1073/pnas.2118539119 PMC9457591

[68] J. M. McCracken, R. S. Weigel, Convergent cross-mapping and pairwise asymmetric inference. Phys. Rev. E **90**, 062903 (2014). 10.1103/PhysRevE.90.062903 25615160

[69] A. T. Clark , Spatial convergent cross mapping to detect causal relationships from short time series. Ecology **96**, 1174–1181 (2015). 26236832 10.1890/14-1479.1

[70] C. W. J. Granger, Investigating causal relations by econometric models and cross-spectral methods. Econometrica **37**, 424–438 (1969).

[71] C. W. J. Granger, Some recent development in a concept of causality. J. Econom. **39**, 199–211 (1988).

[72] T. Hothorn , lmtest: An R Package for Testing Linear Regression Models (Version 0.9–40, CRAN, 2022). https://cran.r-project.org/web/packages/lmtest/lmtest.pdf. Accessed 15 September 2022.

[73] F. Pedregosa , Scikit-learn: Machine learning in Python. J. Mach. Learn. Res. **12**, 2825–2830 (2011).

[74] S. C. Johnson, Heirarchical clustering schemes. Psychometrika **32**, 241–254 (1964). 10.1007/BF02289588 5234703

[75] F. Murtagh, P. Contreras, Algorithms for hierarchical clustering: An overview. WIREs Data Min. Knowl. Discov. **2**, 86–97 (2012).

[76] J. H. Ward Jr., Hierarchical grouping to optimize an objective function. J. Am. Stat. Assoc. **58**, 236–244 (1963).

[77] A. Zeileis, F. Leisch, K. Hornik, C. Kleiber, strucchange: An R package for testing for structural change in linear regression models. J. Stat. Softw. **7**, 1–38 (2002).

[78] J. Bai, P. Perron, Computation and analysis of multiple structural change models. J. Appl. Econom. **18**, 1–22 (2003).

[79] P. Soranno, P. J. Hanly, K. E. Webster, Dataset for: Abrupt changes in algal biomass of thousands of US lakes are related to climate and are more likely in low-disturbance watersheds (v1.0) [Data set]. Zenodo. 10.5281/zenodo.10926306. Deposited 4 April 2024. PMC11892623 39993195

