# Supplementary material for: Abrupt changes in algal biomass of thousands of US lakes are related to climate and are more likely in low-disturbance watersheds
Source: Proc Natl Acad Sci U S A. 2025 Feb 24;122(9):e2416172122. doi: 10.1073/pnas.2416172122 (PMC11892623; doi:10.1073/pnas.2416172122)
Supplement: Supplementary file 1 — Appendix 01 (PDF) [file pnas.2416172122.sapp.pdf]

Supporting information for

Abrupt changes in algal biomass of thousands of US lakes are related to climate and are more likely in low-disturbance watersheds

Patricia A. Soranno, Patrick J. Hanly, Katherine E. Webster, Tyler Wagner, Andrew McDonald, Arnab Shuvo, Erin M. Schliep, Kaitlin L Reinl, Ian M. McCullough, Pang-Ning Tan, Noah R. Lottig, Kendra Spence Cheruvelil

Patricia A. Soranno
E-mail: soranno@msu.edu

This PDF file includes:

Supporting information text
Figs. S1 to S9
Tables S1 to S2
SI References

Supporting Information Text

Representation of Study Lakes

Geographic representation: We assigned lakes to regions of the conterminous US using NEON zones, which are ecoclimatic regions delineated from primarily climate variables (Table S1). Using these regions, our data filtering resulted in sampling rates of a low of 4% to a high of 43% of lakes in a region. This study population represents 17.8% of all lakes in the conterminous US $\geq$ 4 ha, broadly distributed across a wide range of different environmental ecoclimatic variables. Table S2 also shows that we 'sample' lakes similar to their NEON region representation in the full population of lakes except for the Southeast region that is undersampled (4% versus 16% of the full population), Ozarks (4% versus 11%) and, is oversampled in the Great Lakes region (21% relative to the 15% representation in the full population).

Environmental context representation: Equally important as geographical representation is for understanding lake productivity is the representation of study lakes along important ecological ranges for understanding lake productivity. We found that our study population is representative of all lakes in the conterminous US for characteristics such as lake area, lake elevation, % agriculture and forest land use/cover, coarse soil, and groundwater recharge (Fig. S7). We also found that we were not biased in the sampling of lakes within a region by environmental characteristics (Fig. S8). The study lakes tended to be slightly larger than all lakes, which is unsurprising as it is easier to process larger lakes using satellite imagery. However, our study includes smaller lakes than most studies that have quantified temporal changes from satellite data, and our study lakes include the full range of major environmental factors known to control lake productivity. Finally, the slight differences between the study lakes and all lakes do not appear to be ecologically large. This conclusion is based on prior research on lakes in portions of the US demonstrating that even large deviations between sample and full population sample sizes do not significantly alter outcomes of broad-scaled models examining the effects of landscape and climatic features on lake nutrients (1). This analysis also showed that only extremely biased sampling designs result in poorer model performance or conclusions when sample sizes are as large as ours.

Additional Methods and Results for Temporal Patterns Analysis

To assess relationships between the assignment of a lake into one of nine ecological temporal classes and ecological context, we modeled the effect of 32 watershed and lake features on class assignment. We used a lasso and elastic-net regularized generalized linear model (GLMNet) implemented in the R package 'glmnet' (2). We transformed (natural log for non-percentage data when needed and logit generalized to -0.01 to 1.01 for percentage data) and then scaled and standardized each feature. The GLMNet model was a multinomial classification with a grouped penalty (λ) selected through cross-validation. Because the penalty is grouped, features are retained or dropped entirely when predicting temporal class assignment; consequently, 4 of the 32 features tested in the multinomial GLMNet were dropped. To quantify the relative importance of these features on ecological temporal class assignment, we summed the absolute value of the parameter estimates across ecological temporal classes; a higher summed value indicates a higher overall weight of that feature in distinguishing lake ecological temporal class. We found strong associations between many lake and watershed characteristics and the class assignment, supporting our use of the nine classes (Fig. S4).

Supplemental Figures

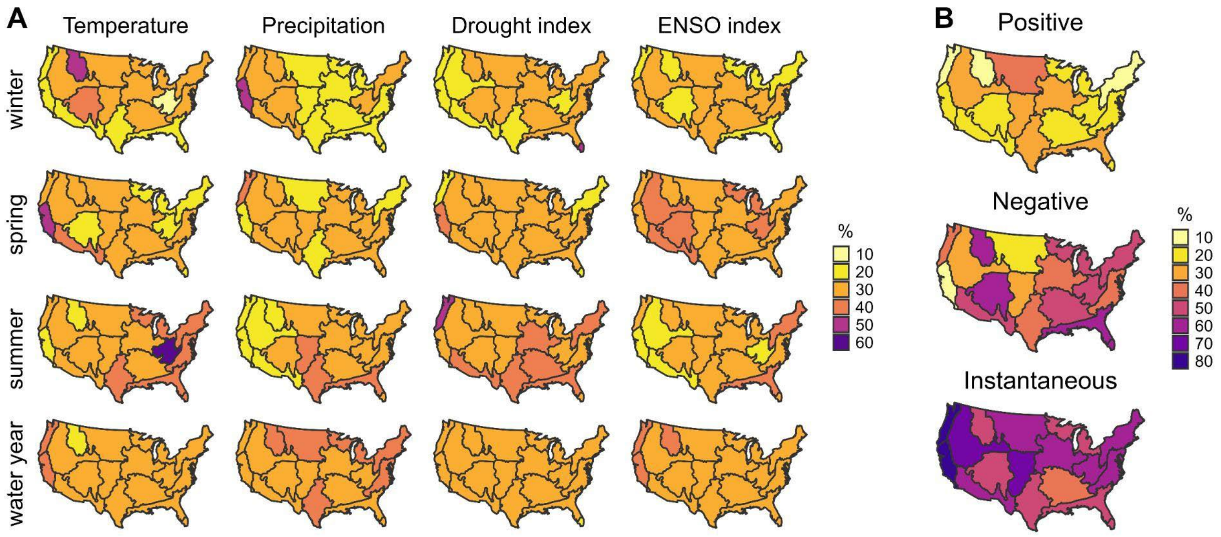

Fig S1. The percent of lakes in a region that are climate causal by climate metric and season. (A) Maps of the percentage of lakes in a NEON region causally related to the climate metric (column) by season (rows). (B) Maps of the percentage of lakes in a NEON region related to temperature for any season for the VAR models only that are Granger causal for either lag-1 positive, lag-1 negative, or instantaneous. Each lake can have one of the lag-1 effects and an instantaneous effect, so the categories are not mutually exclusive.

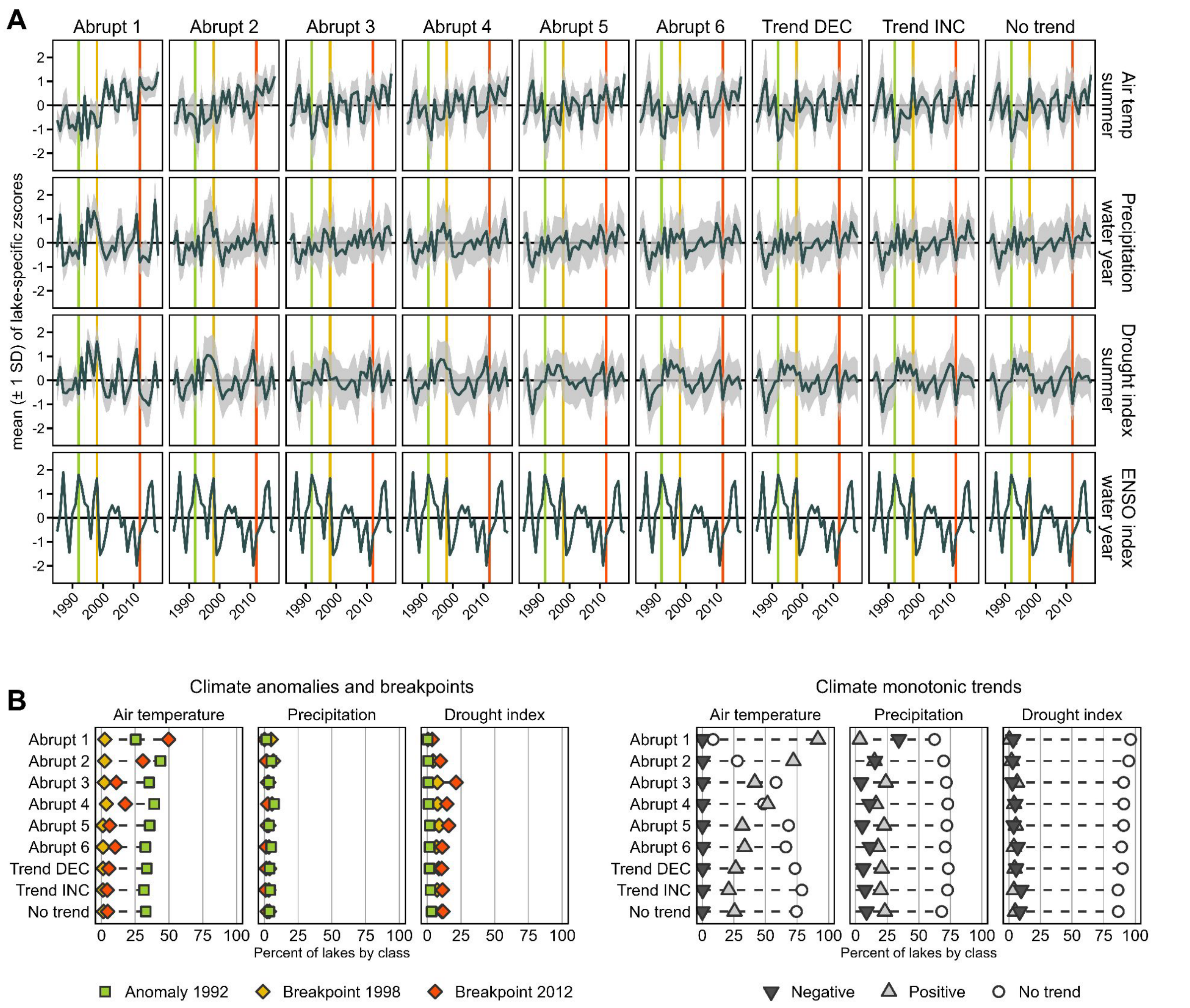

Fig. S2. Time series of climate metrics by ecological temporal class. (A) Plots of the mean climate metric z-scores by ecological temporal class and 1 standard deviation from the mean (gray shading), except for ENSO, which is a single value for all lakes. (B) Dot plot showing the percentage of climate time series by class that showed a breakpoint or an anomaly in the same years shown in Figure 3 (lower left panels) or monotonic trends (right panels) for any season (multiple seasons with the same breakpoint are only counted once).

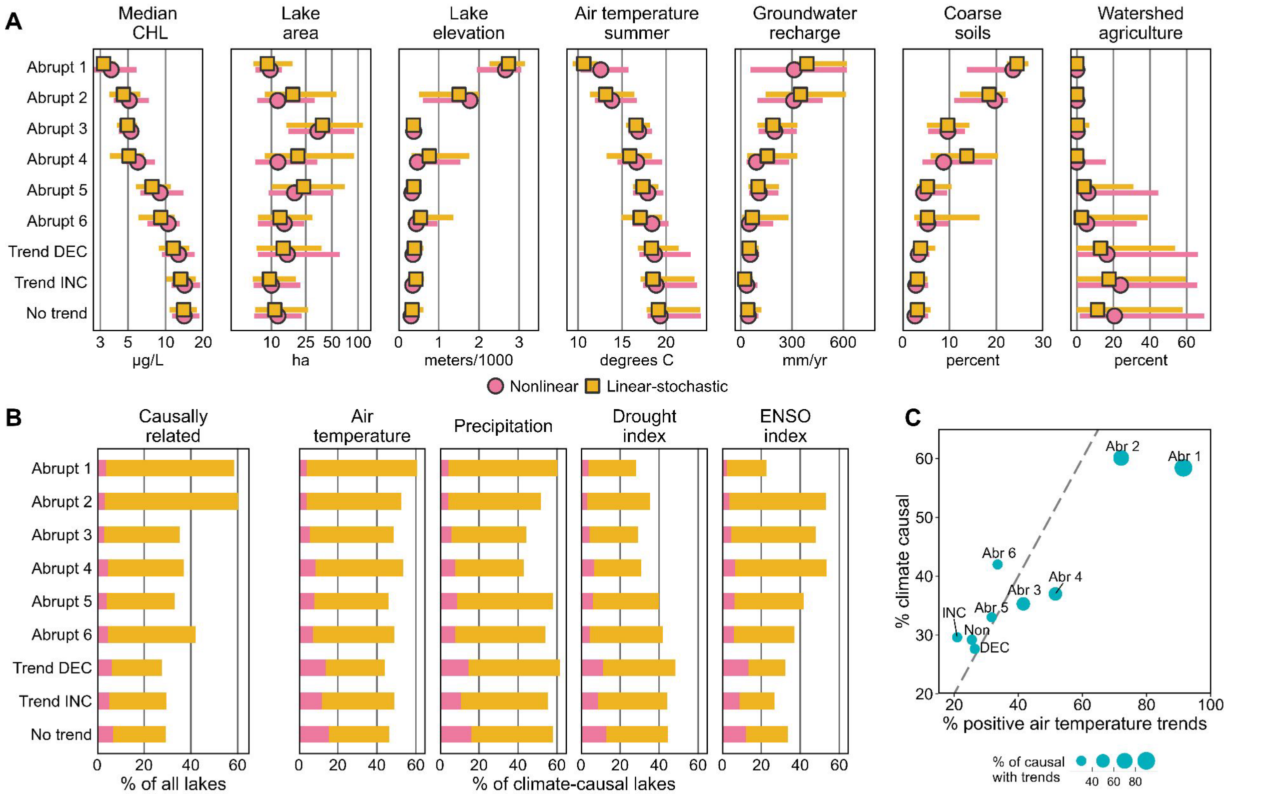

Fig. S3. Characteristics of lakes by ecological temporal class. (A) Median CHL and environmental characteristics of climate-causal lakes by model type (lines indicate the IQR). (B) Percentage of all lakes within an ecological temporal class that is climate-causal by model type (nonlinear vs linear-stochastic). (C) The percent of climate causal lakes versus the percent of lakes with monotonic trends in air temperature for each class. The size of the bubbles indicates where both criteria are met; the dashed line is the 1:1 relationship. INC is the Trend INC lake class, DEC is the Trend DEC lake class, and NON is the No trend lake class.

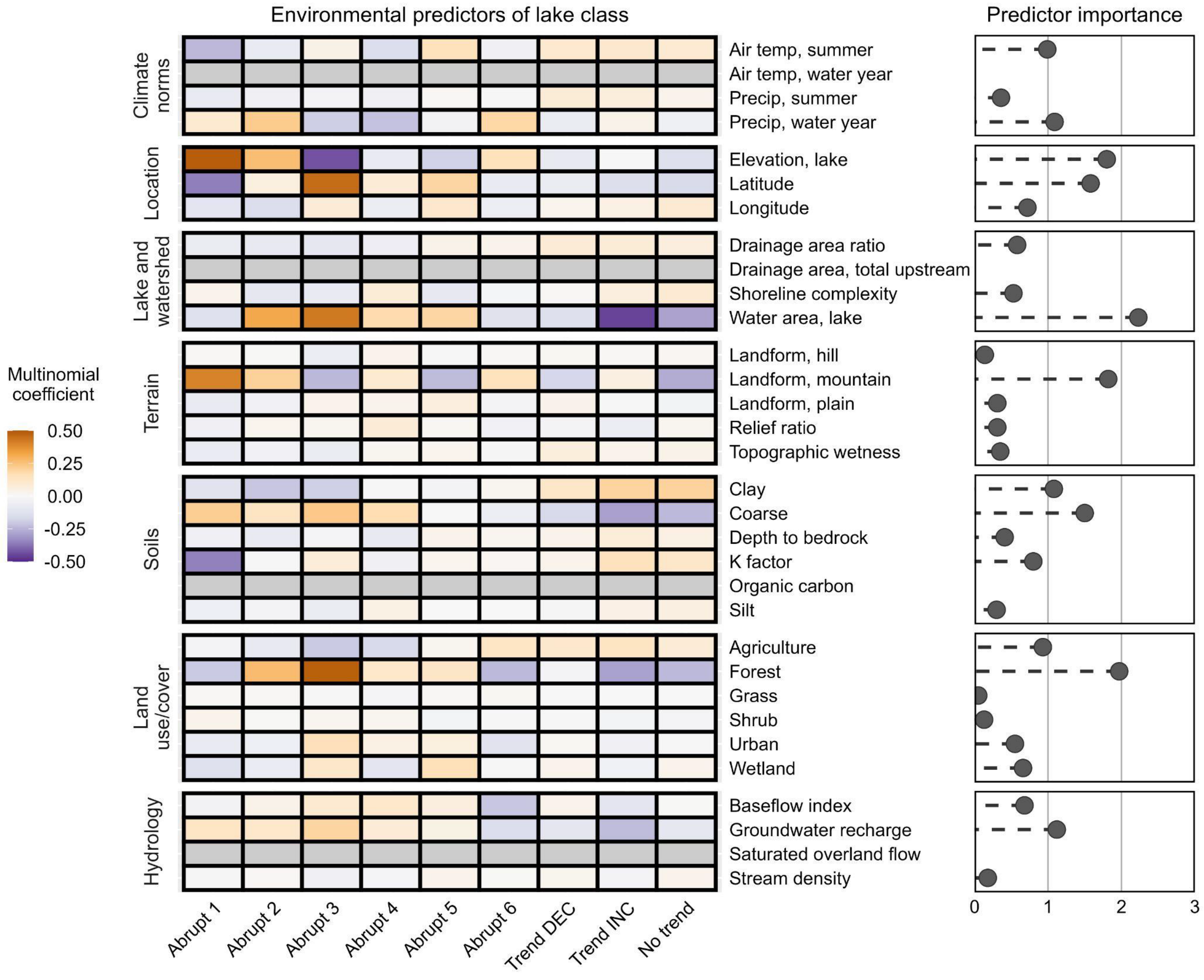

Fig. S4. Relationship between ecological temporal cluster and natural and human environmental context. (A) A heatmap showing the positive (brown) to negative (purple) multinomial coefficients from GLMnet models analyzing relationships between the assignment of a lake to an ecological temporal class and common natural and human environmental characteristics known to affect lake CHL. (B) Plot of the absolute value of the summed multinomial coefficient as a measure of overall importance for each characteristic across all of the classes.

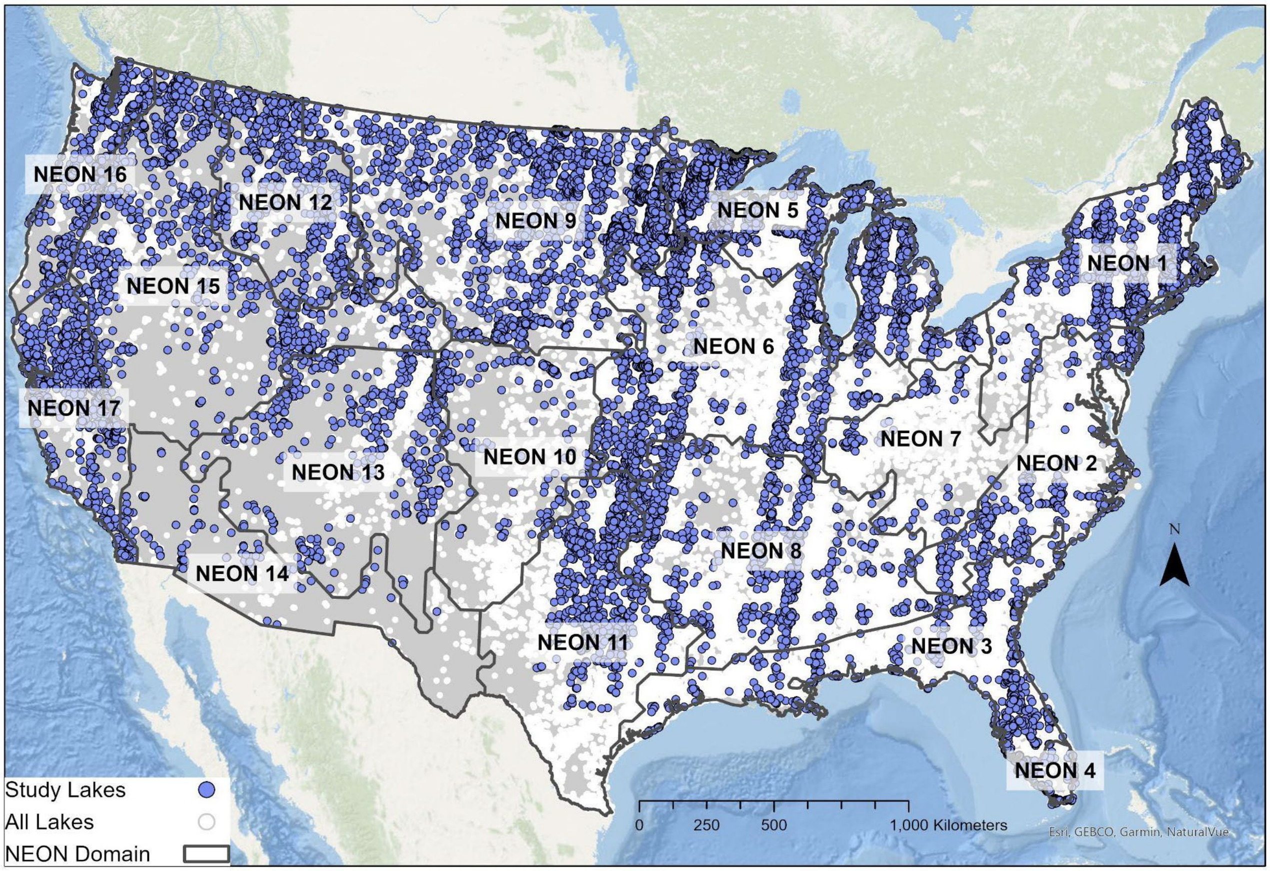

Fig S5. Map showing the location of the 24,452 study lakes with 34-yr CHL time series by NEON region. The 24,452 lakes appear to be aligned in 'bands' due to increased imagery available for lakes with overlapping scene paths of Landsat satellites, which improves the likelihood of cloud-free images.

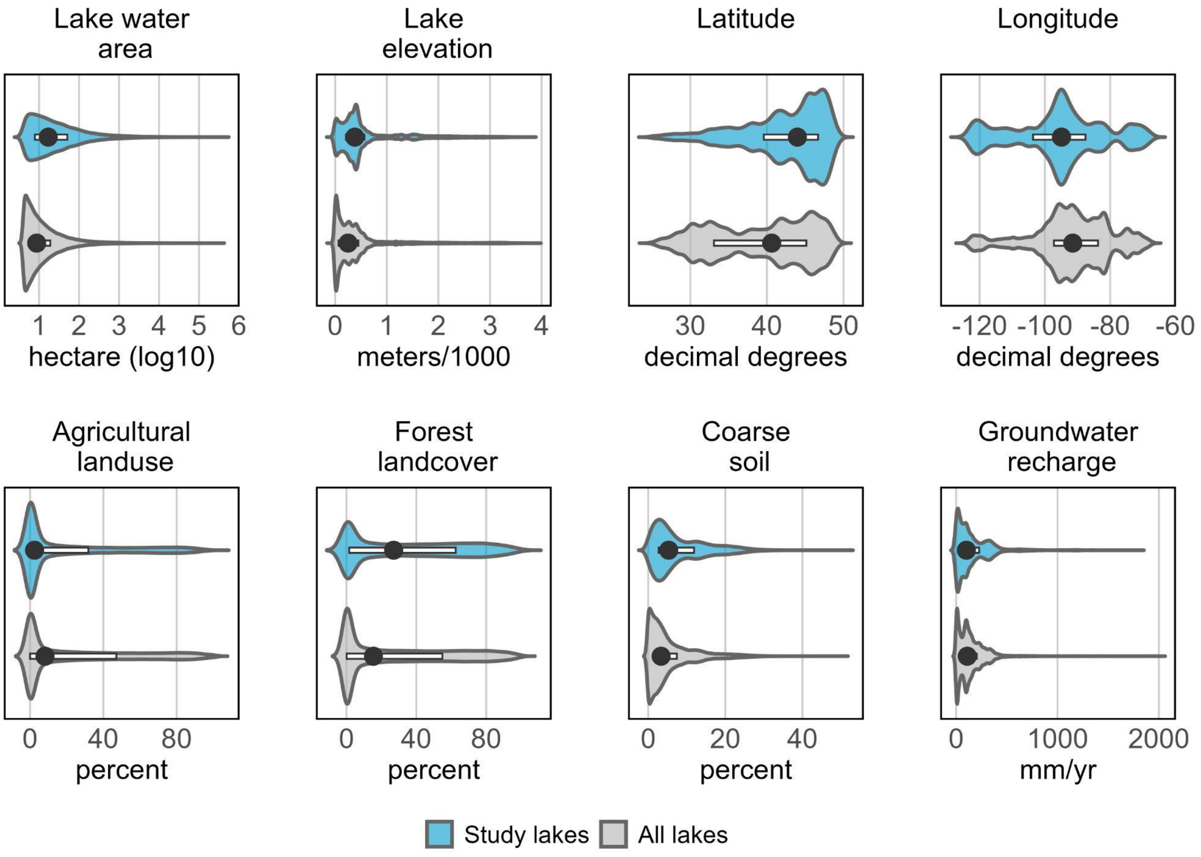

Fig S6. Comparison of study lakes to all possible lakes ≥ 4 ha in the US The violin plots show all data for the two groups of lakes; the black dots are the median values; and, the boxes within the violin plots are the interquartile ranges. The study lakes are included in the all-lake population derived from the LAGOS lake population ≥ 4 ha.

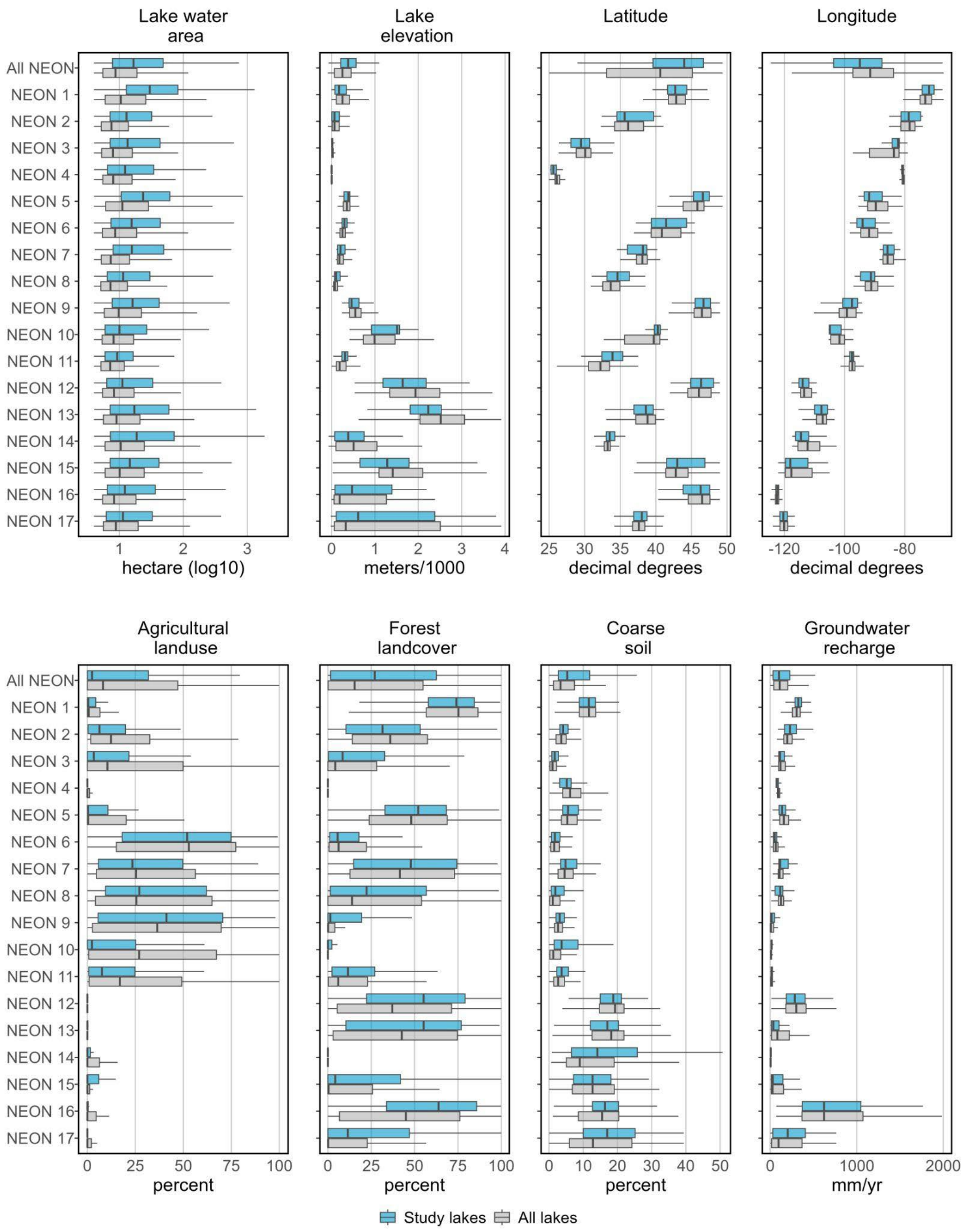

Fig S7. Comparison of study lakes to all possible lakes ≥ 4 ha in the US by NEON region. Boxplots (showing the IQR and whisker extending to 1.5 times the upper and lower 75th and 25th quartiles).

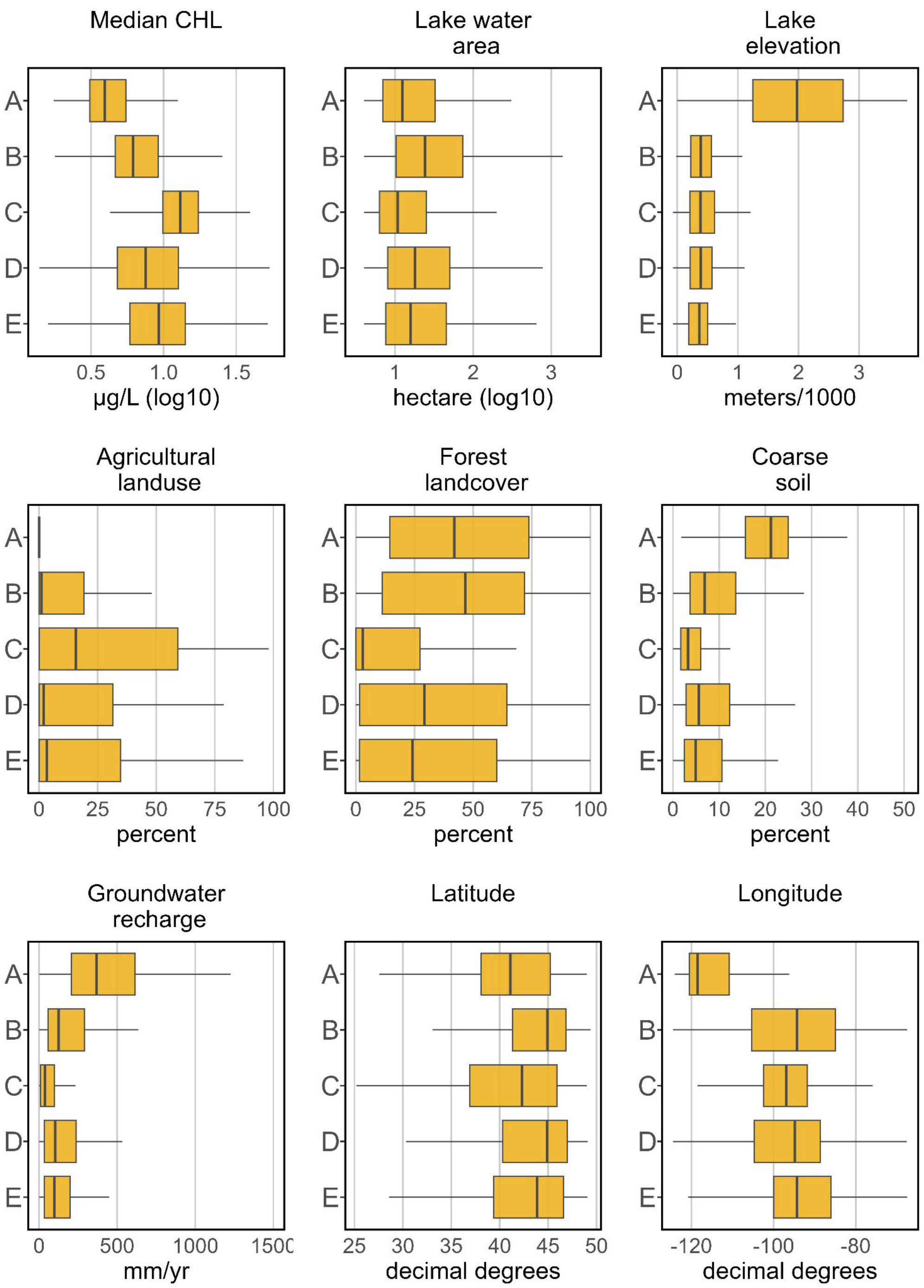

Fig S8. Boxplots showing ranges of environmental context characteristics for each climate response type of lakes (defined in Figure 5) that are more or less likely to be causally related to climate. Lakes range in climate responsiveness from the most responsive type A, which are abrupt lakes, whilst E lakes had time series that were unpredictable and not related to climate.

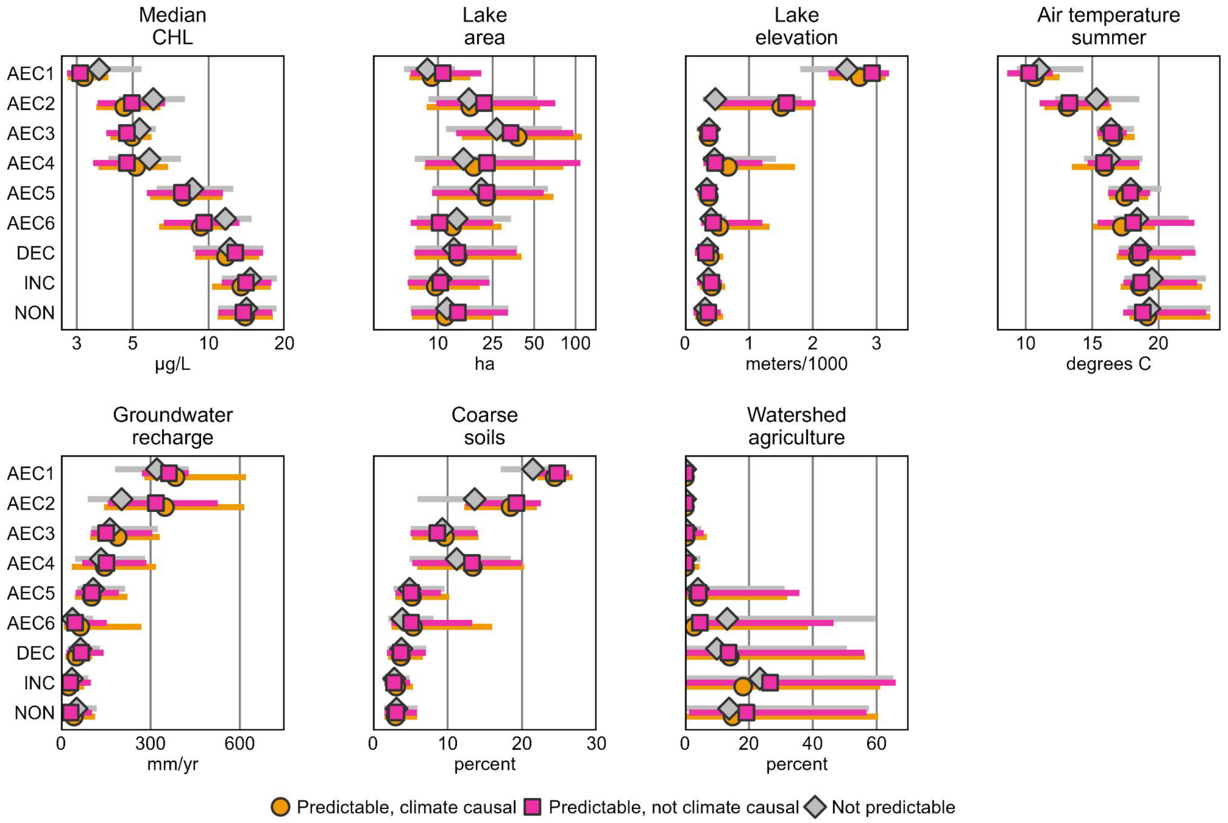

Fig S9. Medians and the interquartile ranges (lines) for environmental context characteristics for lake ecological classes that are plotted by statistical property and causality. Long-term median CHL and lake area are plotted on a log scale. Ecological temporal class labels are as for Fig. 3, except that AEC = Abrupt, and DEC and INC indicate classes with either decreasing (DEC) or increasing (INC) monotonic trends, and NON indicates no pattern. Statistical properties are defined in Fig. 2A.

Supplemental Tables

Table S1. The percent (and number) of climate-causal lake CHL time series at $p < 0.05$. (A) Results for linear-stochastic CHL time series using VAR models to estimate Granger causality of a climate metric on a lake CHL time series by season (previous winter, spring, summer, or water year) and by lag (positive lag 1, negative lag 1, or instantaneous). (B) Results for nonlinear CHL time series using CCM models to estimate causality of a climate metric on a lake CHL time series by season (previous winter, spring, summer, or water year). CCM models do not estimate lags or directionality of effects due to the complexity of nonlinear relationships. Because a lake CHL time series can be causally linked to more than one climate metric, season, or lag, the percentages do not add to the total number of significant causal relationships.

A. Linear-stochastic (G)

Percent

Climate variable	Lag	Previous Winter	Spring	Summer	Water Year	*Any season*
**Temperature**	Positive lag	1.6%	3.1%	3.0%	2.3%	*7.9%*
	Negative lag	7.5%	2.7%	6.6%	5.9%	*16.1%*
	Instantaneous	7.1%	9.5%	11.1%	11.0%	*26.7%*
	Any temp metric	***16.0%***	***15.0%***	***19.9%***	***18.5%***	***45.8%***
**Precipitation**	Positive lag	2.3%	3.3%	2.8%	2.3%	*9.2%*
	Negative lag	6.7%	5.2%	6.1%	8.1%	*19.8%*
	Instantaneous	6.8%	7.6%	9.3%	15.6%	*28.7%*
	Any PPT metric	***15.2%***	***15.6%***	***17.6%***	***24.7%***	***51.3%***
**ENSO index**	Positive lag	2.8%	3.7%	6.7%	3.8%	*11.2%*
	Negative lag	2.3%	2.0%	1.0%	1.4%	*4.4%*
	Instantaneous	8.8%	15.6%	11.0%	16.4%	*29.2%*
	Any MEI metric	***13.6%***	***20.9%***	***18.4%***	***21.1%***	***38.1%***
**Drought index**	Positive lag	3.4%	2.5%	2.8%	2.6%	*6.4%*
	Negative lag	3.8%	5.2%	6.9%	5.3%	*10.9%*
	Instantaneous	8.0%	10.1%	14.1%	11.1%	*23.9%*
	Any PHDI metric	***14.6%***	***17.0%***	***22.3%***	***18.2%***	***36.1%***

Number (N)

Climate variable	Lag	Previous Winter	Spring	Summer	Water Year	*Any season*
**Temperature**	Positive lag	113	227	218	167	*574*
	Negative lag	542	197	480	425	*1167*
	Instantaneous	512	687	801	794	*1930*
	Any temp metric	***1160***	***1083***	***1443***	***1337***	***3317***
**Precipitation**	Positive lag	170	239	203	168	*665*
	Negative lag	483	377	445	588	*1435*
	Instantaneous	495	553	670	1128	*2076*
	Any PPT metric	***1100***	***1127***	***1272***	***1788***	***3717***
**ENSO index**	Positive lag	206	267	485	272	*813*
	Negative lag	170	148	74	101	*315*
	Instantaneous	634	1130	800	1186	*2115*
	Any MEI metric	***982***	***1513***	***1335***	***1531***	***2760***
**Drought index**	Positive lag	247	184	205	188	*466*
	Negative lag	272	374	497	384	*791*
	Instantaneous	581	731	1018	803	*1728*
	Any PHDI metric	***1059***	***1233***	***1614***	***1317***	***2617***
**Any Climate Granger-causal (Total):**						***N = 7240***

B. Nonlinear (CCM)

Percent

Climate variable		Previous Winter	Spring	Summer	Water Year	*Any season*
**Temperature**	*NA*	27.2%	30.3%	29.4%	28.1%	*65.3%*
**Precipitation**	*NA*	28.7%	28.6%	27.8%	26.0%	*67.8%*
**ENSO index**	*NA*	30.2%	28.5%	27.3%	29.0%	*53.9%*
**Drought index**	*NA*	28.1%	28.0%	26.5%	27.7%	*50.8%*

Number (N)

Climate variable		Previous Winter	Spring	Summer	Water Year	*Any season*
**Temperature**	*NA*	302	337	327	313	*726*
**Precipitation**	*NA*	319	318	309	289	*754*
**ENSO index**	*NA*	336	317	304	323	*599*
**Drought Index**	*NA*	312	311	295	308	*565*
**Any Climate CCM-causal (Total):**						***N = 1112***

Table S2. The number and percentage of study lakes compared to all lakes ≥ 4 ha by NEON region.

NEON Domain	NEON Name	Study Lakes N	Study Lakes Percent	All Lakes N	All Lakes Percent	Study Lakes % of All lakes
1	Northeast	2878	11.8	13641	9.9	21.1
2	Mid Atlantic	597	2.4	6521	4.7	9.2
3	Southeast	1082	4.4	21444	15.6	5.0
4	Atlantic Neotropical	154	0.6	1625	1.2	9.5
5	Great Lakes	5118	20.9	20806	15.1	24.6
6	Prairie Peninsula	2119	8.7	11476	8.3	18.5
7	Appalachians / Cumberland Plateau	129	0.5	3257	2.4	4.0
8	Ozarks Complex	1027	4.2	15203	11.1	6.8
9	Northern Plains	4578	18.7	18971	13.8	24.1
10	Central Plains	587	2.4	2126	1.5	27.6
11	Southern Plains	961	3.9	7990	5.8	12.0
12	Northern Rockies	1081	4.4	2724	2.0	39.7
13	Southern Rockies / Colorado Plateau	495	2.0	1944	1.4	25.5
14	Desert Southwest	120	0.5	477	0.3	25.2
15	Great Basin	1533	6.3	4348	3.2	35.3
16	Pacific Northwest	799	3.3	2111	1.5	37.8
17	Pacific Southwest	1194	4.9	2801	2.0	42.6

SI References

1. P. A. Soranno, *et al.*, Ecological prediction at macroscales using big data: Does sampling design matter? *Ecol. Appl.* **30**, e02123 (2020).

2. J. Friedman, *et al.*, glmnet: Lasso and Elastic-Net Regularized Generalized Linear Models. (2023). Deposited 22 August 2023.
